# Distinct mechanisms of allopregnanolone and diazepam underlie neuronal oscillations and differential antidepressant effect

**DOI:** 10.3389/fncel.2023.1274459

**Published:** 2024-01-08

**Authors:** Keiko Takasu, Yosuke Yawata, Ryoichi Tashima, Hiroyuki Aritomi, Shinji Shimada, Tsukasa Onodera, Teruhiko Taishi, Koichi Ogawa

**Affiliations:** ^1^Laboratory for Drug Discovery and Disease Research, Shionogi Pharmaceutical Research Center, Shionogi & Co., Ltd., Osaka, Japan; ^2^Shionogi TechnoAdvance Research, Osaka, Japan

**Keywords:** neuroactive steroid, benzodiazepine, extrasynaptic GABA_A_ receptor, social defeat stress model, antidepressant-like effect, theta activity, basolateral amygdala

## Abstract

The rapid relief of depressive symptoms is a major medical requirement for effective treatments for major depressive disorder (MDD). A decrease in neuroactive steroids contributes to the pathophysiological mechanisms associated with the neurological symptoms of MDD. Zuranolone (SAGE-217), a neuroactive steroid that acts as a positive allosteric modulator of synaptic and extrasynaptic δ-subunit-containing GABA_A_ receptors, has shown rapid-onset, clinically effective antidepressant action in patients with MDD or postpartum depression (PPD). Benzodiazepines, on the other hand, act as positive allosteric modulators of synaptic GABA_A_ receptors but are not approved for the treatment of patients with MDD. It remains unclear how differences in molecular mechanisms contribute to the alleviation of depressive symptoms and the regulation of associated neuronal activity. Focusing on the antidepressant-like effects and neuronal activity of the basolateral amygdala (BLA) and medial prefrontal cortex (mPFC), we conducted a head-to-head comparison study of the neuroactive steroid allopregnanolone and the benzodiazepine diazepam using a mouse social defeat stress (SDS) model. Allopregnanolone but not diazepam exhibited antidepressant-like effects in a social interaction test in SDS mice. This antidepressant-like effect of allopregnanolone was abolished in extrasynaptic GABA_A_ receptor δ-subunit knockout mice (δko mice) subjected to the same SDS protocol. Regarding the neurophysiological mechanism associated with these antidepressant-like effects, allopregnanolone but not diazepam increased theta oscillation in the BLA of SDS mice. This increase did not occur in δko mice. Consistent with this, allopregnanolone potentiated tonic inhibition in BLA interneurons via δ-subunit-containing extrasynaptic GABA_A_ receptors. Theta oscillation in the mPFC of SDS mice was also increased by allopregnanolone but not by diazepam. Finally, allopregnanolone but not diazepam increased frontal theta activity in electroencephalography recordings in naïve and SDS mice. Neuronal network alterations associated with MDD showed decreased frontal theta and beta activity in depressed SDS mice. These results demonstrated that, unlike benzodiazepines, neuroactive steroids increased theta oscillation in the BLA and mPFC through the activation of δ-subunit-containing GABA_A_ receptors, and this change was associated with antidepressant-like effects in the SDS model. Our findings support the notion that the distinctive mechanism of neuroactive steroids may contribute to the rapid antidepressant effects in MDD.

## Introduction

Major depressive disorder (MDD) is a psychiatric disorder characterized by an episode of core depressive symptoms lasting at least 2 weeks, including pervasive low mood or loss of interest or pleasure in normally enjoyable activities (American Psychiatric Association, 2013). Approved drugs for MDD take at least 4–8 weeks to show efficacy (Rush et al., 2006). A delayed onset of response has been associated with decreased treatment adherence, leading to incomplete remission and relapse (Habert et al., 2016). Rapid relief of depressive symptoms through effective treatment is therefore an important medical requirement for improving the quality of life of patients with MDD.

Brexanolone (allopregnanolone) and zuranolone (SAGE-217) are neuroactive steroids that are used clinically as fast-acting antidepressant agents; these compounds act on synaptic and extrasynaptic GABA_A_ receptors (Edinoff et al., 2021; Ali et al., 2023; Kato et al., 2023). Previous studies have reported decreased plasma or brain concentrations of the endogenous neuroactive steroid allopregnanolone in patients with MDD, which correlates with the severity of depressive symptoms (Uzunova et al., 1998; Agis-Balboa et al., 2014). Increased plasma concentrations of allopregnanolone with antidepressant treatment are associated with the relief of depressive symptoms (Romeo et al., 1998). Therefore, the potentiation of GABA_A_ receptors by increasing neuroactive steroids may contribute to fast-acting antidepressant effects in patients with MDD. In contrast, benzodiazepines also act as positive allosteric modulators of the GABA_A_ receptor but are not approved for the treatment of patients with MDD (Lim et al., 2020). The differences in efficacies in patients with MDD may be caused by distinct molecular mechanisms. There are two subtypes of GABA_A_ receptors, namely, synaptic and extrasynaptic GABA_A_ receptors. Most δ-subunit-containing GABA_A_ receptors seem to be purely extrasynaptic, although there are various subunit compositions (Brickley and Mody, 2012). In addition to the difference of the subunits, neuroactive steroids specifically enhance a tonic inhibitory conductance that is mediated by δ-subunit-containing GABA_A_ receptors at lower concentrations, and then they also start to potentiate phasic inhibition at higher concentrations (Stell et al., 2003; Belelli and Lambert, 2005; Farrant and Nusser, 2005; Brickley and Mody, 2012; Martinez et al., 2017). It has also been reported that neuroactive steroids potentiate both synaptic and extrasynaptic (δ-subunit-containing) GABA_A_ receptors by binding to α and β subunits, which are common to both types of receptors (Sigel, 2002; Hosie et al., 2006; Carver and Reddy, 2013; Nuss, 2015; Alvarez and Pecci, 2018). In contrast, benzodiazepines primarily activate synaptic GABA_A_ receptors by binding to α and γ subunits (Carver and Reddy, 2013; Nuss, 2015). Therefore, based on differences in the binding and activation mode, δ-subunit-containing GABA_A_ receptors could largely contribute to differences in efficacies in patients with MDD. However, it remains unclear how differences in molecular mechanisms contribute to alleviating depressive symptoms and modulating neuronal network activity.

The dysregulation of neural network activity in brain regions is associated with depressive symptoms in patients with MDD (Drevets, 2000; Phillips et al., 2003; Northoff et al., 2011). The amygdala is one of the regions with high concentrations of neuroactive steroids involved in the pathology of MDD (Bixo et al., 2018). Altered neuronal activity between the amygdala and its regulated brain regions, including the prefrontal cortex, in MDD patients has been implicated in stress vulnerability and negative emotions (Drevets, 2000; Phillips et al., 2003). Neuroactive steroids regulate emotional cognition by altering the functional connectivity of the amygdala and frontal cortex (Sripada et al., 2014). Recent preclinical studies have demonstrated that increased basolateral amygdala (BLA) theta activity via potentiation of δ-subunit-containing GABA_A_ receptors on interneurons can contribute to the antidepressant effects of neuroactive steroids in chronic unpredictable stress model mice (Antonoudiou et al., 2022; Luscher et al., 2023; Walton et al., 2023). The benzodiazepine diazepam also acts on the amygdala but does not increase theta activity in naïve mice (Antonoudiou et al., 2022). BLA theta activity is therefore thought to be related to differential antidepressant effects between neuroactive steroids and benzodiazepines. However, there have been no reports of direct comparisons between neuroactive steroids and benzodiazepines under identical conditions using a preclinical model of depression.

In the present study, we used the social interaction test (SIT) in a mouse social defeat stress (SDS) model and collected *in vivo* recordings of oscillation in the BLA and medial prefrontal cortex (mPFC) to assess differences in the effect of allopregnanolone, a neuroactive steroid, and diazepam, a benzodiazepine drug, by direct comparison. Allopregnanolone, but not diazepam, potentiates δ-subunit-containing GABA_A_ receptors in BLA interneurons and increases theta oscillation in the BLA and mPFC. This mechanism may contribute to the development of antidepressant-like effects in depressed SDS mice.

## Materials and methods

### Animals

Experiments were performed using C57BL/6 J Jcl mice, GABA_A_ receptor δ-subunit knockout mice (δΚΟ mice, Gabrd^−/−^ mice), and Crl:CD1 (ICR) retired mice. ICR mice (male) and Gabrd^−/−^ mice (male) were purchased from Jackson Laboratory. Gabrd^−/−^ mice are homozygous null for Gabrd (Mihalek et al., 1999). The breeding of Gabrd^−/−^ mice was approved by the Animal Care and Use Committee of Shionogi Research Laboratories and was in accordance with the Association for Assessment and Accreditation of Laboratory Animal Care (AAALAC) International guidelines. C57BL/6 J Jcl mice (male) were purchased from CLEA Japan Inc. and were used as wild-type controls. The body weights of C57BL/6 J Jcl mice were 20–30 g. Mice aged 2–4 months were used. The mice were housed under controlled temperature and humidity with a 12/12-h light/dark cycle (light from 8:00 to 20:00). Less than three mice were housed in a cage (W 235 mm, D 353 mm, H 160 mm) with a paper-chip bedding (SLC Japan, Inc.) and Nesting Sheets™ (Bio-Serv, United States) as environmental enrichment. Mice were allowed *ad libitum* access to food (CE-2, CLEA Japan, Inc.) and clean water (filtered at 5 mm from Toyonaka City, Japan) under SPF conditions. All procedures were approved by the Animal Care and Use Committee of Shionogi Research Laboratories, Osaka, Japan. Electrophysiological assessments were performed according to the AAALAC International guidelines.

### Drugs

Allopregnanolone was purchased from Toronto Research Chemicals (Canada). Diazepam and methyl cellulose were purchased from Fujifilm (Japan). Escitalopram was purchased from MedChemExpress (United States). Hydroxypropyl-β-cyclodextrin was purchased from Tokyo Chemical Industry (Japan). Allopregnanolone and diazepam were dissolved in 15% hydroxypropyl-β-cyclodextrin in distilled water. Escitalopram was dissolved in methyl cellulose. Allopregnanolone and diazepam were administered intraperitoneally. Escitalopram was administered by oral gavage.

### Preparation of the SDS model

**Figure 1:**
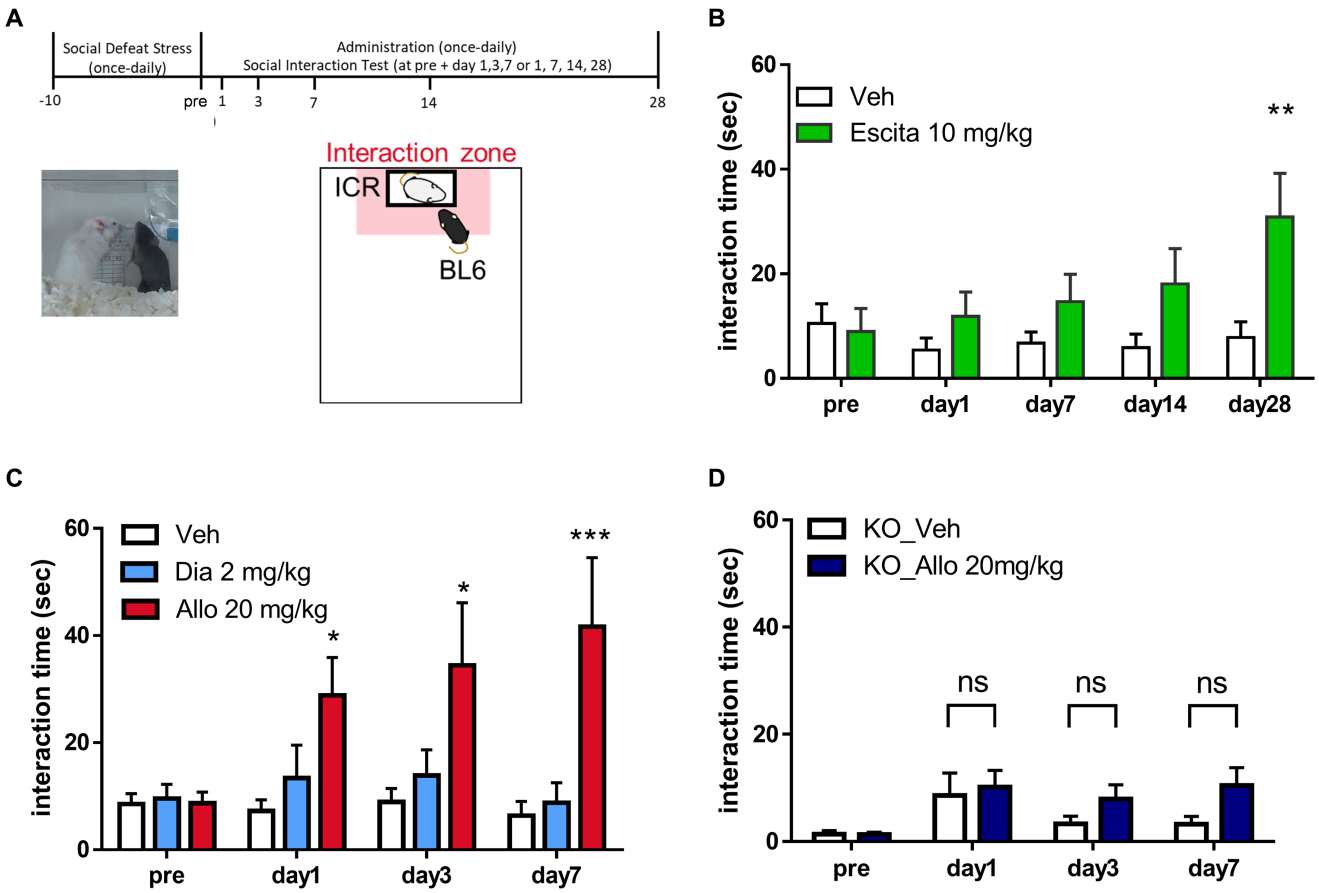
Antidepressant-like effects of allopregnanolone, in contrast to diazepam. **(A)** Behavioral paradigm of antidepressant assessment in the social interaction test (SIT) of the social defeat stress (SDS) model. A C57BL/6 J Jcl mouse was defeated by a larger ICR mouse for 10 days. SIT was conducted for the purpose of identifying depression-like mice and assessing antidepressant effects following drug administration. Data are expressed as the interaction time that each mouse spent in the direct interaction zone (close to the box with the ICR mouse, depicted in the pale red zone). **(B)** Time course of the interaction time before and after the administration of vehicle (Veh, white column) and a selective serotonin reuptake inhibitor escitalopram (Escita, green column) in wild-type mice with the SDS model. Data are represented as the mean ± SEM. ^*^p = 0.003, *n* = 9–10 mice, Sidak’s test. **(C)** Time course of the interaction time before and after administration of the vehicle (Veh, white column), the benzodiazepine diazepam (Dia, blue column), and the neuroactive steroid allopregnanolone (Allo, red column) in wild-type mice with the SDS model. Data are represented as the mean ± SEM. ^*^p = 0.038 at day 1, ^*^p = 0.011 at day 3, ^***^p = 0.0003 at day 7, *n* = 13 mice, Tukey’s test. **(D)** Time course of the interaction time before and after the administration of Veh (white column) and Allo (dark blue column) in GABA_A_ receptor δ-subunit knockout mice (δko mice) with the SDS model. Data are represented as the mean ± SEM. *p* = 0.985 at day 1, *p* = 0.573 at day 3, *p* = 0.163 at day 7, *n* = 20 mice, Sidak’s test.

A C57BL/6 J Jcl mouse was defeated by a larger stranger ICR mouse. Each individual defeat lasted 10 min. If the mice developed severe injuries or extreme weakness during the 10-day defeat procedure, they were euthanized at the veterinarian’s discretion (Figure 1A). After the 10-day defeat procedure, SIT was conducted to select the mice with depression-like behavior with a series of 3 sequential tests: (1) Each C57BL/6 J Jcl mouse was placed in an open-field chamber (40 × 30 × 20 cm) with an empty wire mesh box at one end, and the mouse was allowed to freely explore the chamber for 150 s while being recorded on video. This session was defined as test 1. (2) After that day, the C57BL/6 J Jcl mouse was placed in an open-field chamber where a novel male ICR mouse was enclosed in a wire mesh box at one end, and the mouse was allowed to freely explore the chamber for 150 s while being recorded on video. This session was defined as test 2. (3) After that day, the same test (test 2) was conducted. This session was defined as test 3. In each test, the time that each mouse spent in the interaction zone (around the wire mesh box with or without the ICR mouse) was calculated by tracking the mass of the mouse with the EthoVision XT video-tracking system (Noldus Information Technology, Wageningen, The Netherlands). The times in tests 1, 2, and 3 were defined as direct SIT time 1 (dSIT1), direct SIT time 2 (dSIT2), and direct SIT time 3 (dSIT3), respectively. A mouse whose dSIT2 and dSIT3 were both lower than dSIT1 was regarded as a depression-like mouse. Depression-like mice were used for further studies, including the SIT, the tail suspension test (TST), a patch-clamp assay in BLA slices, and *in vivo* electrophysiological studies with local field potential (LFP) and electroencephalogram (EEG) recordings.

### Social interaction test

In depression-like mice, the SIT was conducted to evaluate the efficacy of the test substance. Each depression-like C57BL/6 J Jcl mouse was placed in an open-field chamber where a novel male ICR mouse was enclosed in a wire mesh box at one end, and the mouse was allowed to freely explore the chamber for 150 s while being recorded on video. The time that each mouse spent in the direct interaction zone (close to the wire mesh box with the ICR mouse) was calculated by tracking the center of mass of the mouse with an EthoVision XT video-tracking system (Figure 1A). A different ICR mouse was used for each trial to reduce the influence of habituation on social behavior. Allopregnanolone, diazepam, or vehicle was intraperitoneally administered once daily for 7 days. The day when dSIT3 was measured was defined as “pre.” The day of the initial injection was defined as Day 1. In the direct comparison study between allopregnanolone and diazepam, SIT at days 1, 3, and 7 was conducted approximately 30 min after the injection of the drug. The data represent the time that each mouse spent in the direct interaction zone. Analysis was conducted using Tukey’s test for multiple groups and using Sidak’s test for two groups. A value of *p* < 0.05 was considered statistically significant.

### Tail suspension test

In mice with depression-like behavior, the TST was conducted to evaluate the efficacy of the test substance. Each depression-like C57BL/6 J Jcl mouse was suspended in a chamber for 10 min. The time that each mouse spent immobile was calculated by tracking the center of mass of the mouse with an EthoVision XT video-tracking system. In the depression-like mice after SIT for 7 days and the following washout periods for 5–10 days, a tail suspension test was conducted. Allopregnanolone, diazepam, or vehicle was intraperitoneally administered approximately 30 min prior to the test. The data represent the immobility time for each mouse over 8 min. Analysis was conducted using Dunnett’s test. A value of *p* < 0.05 was considered statistically significant.

### Open field test for the assessment of locomotor activity

To identify non-sedative doses of allopregnanolone and diazepam, locomotor activity was assessed in naïve mice. Immediately after the injection of allopregnanolone or diazepam, the C57BL/6 J Jcl mouse was placed in an open-field chamber (40 × 30 × 20 cm), and the mouse was allowed to freely explore the chamber for 40 min while being recorded on video. The distance that each mouse traveled in the chamber was calculated by tracking the mass of the mouse with an EthoVision XT video-tracking system. The data represent the distance that each mouse moved in the chamber for 40 min. Analysis was conducted using Dunnett’s test. A *p-*value of <0.05 was considered statistically significant.

### Open field test for the assessment of the anxiolytic effects of diazepam

To evaluate the anxiolytic effects of diazepam, an open-field device (SCANET MV-40, MELQUEST, Japan) was used. Each mouse was placed in the device 30 min after the injection of diazepam. The time that each mouse stayed in a 30 cm square area in the center area of the device (45× 45 × 12 cm) and the movement in the chamber were measured by the number of squares crossed with the four paws for 10 min. The data represent the total time that each mouse stayed in the center area of the chamber for 10 min. Analysis was conducted using Dunnett’s test. A *p-*value of <0.05 was considered statistically significant.

### Patch clamp assay in BLA slices

#### Preparation of BLA Slices

The mice were euthanized by cervical dislocation. The brains were then quickly removed and placed in ice-cold, low-sodium artificial CSF (cerebrospinal fluid) containing 100 mM choline-Cl, 13 mM NaCl, 3 mM KCl, 1 mM NaH_2_PO_4_, 25 mM NaHCO_3_, 11 mM D-glucose, 1 mM CaCl_2_, and 5 mM MgCl_2_ (pH 7.4 after bubbling with 95% O_2_ and 5% CO_2_). Coronal slices (300 μm thick) were prepared using a vibratome (VT1200S, Leica, Germany) and then maintained for at least 60 min in standard artificial CSF containing 113 mM NaCl, 3 mM KCl, 1 mM NaH_2_PO_4_, 25 mM NaHCO_3_, 11 mM d-glucose, 2 mM CaCl_2_, and 1 mM MgCl_2_ (pH 7.4, after bubbling with 95% O_2_ and 5% CO_2_ at 30–32°C). Slices were transferred to a recording chamber mounted on the stage of a microscope (BX51WI, Olympus, Japan) and superfused with standard artificial CSF (flow rate of 2.5 mL min^−1^ at 30–32°C).

#### Patch-clamp recording

**Figure 2:**
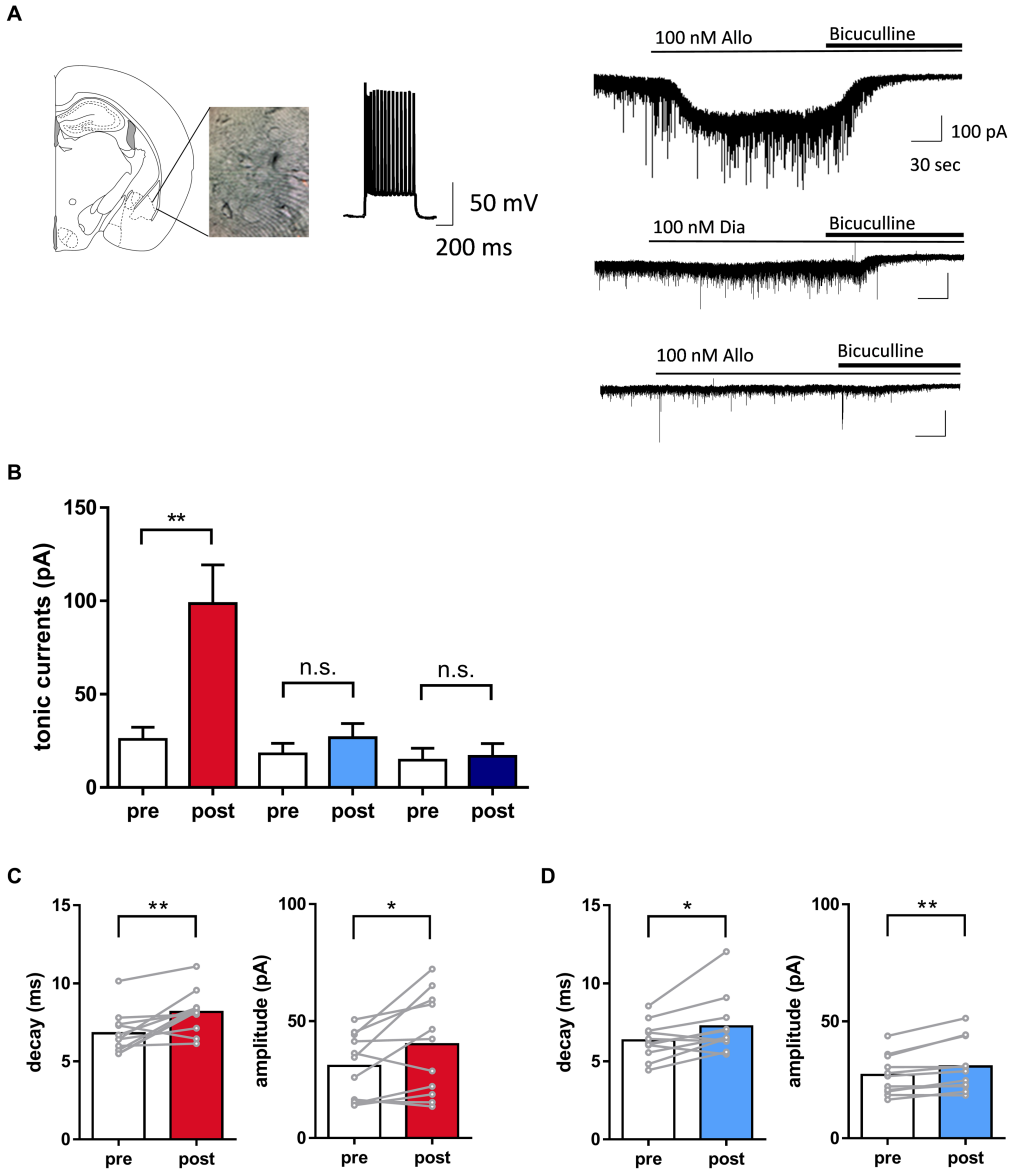
Distinct potentiation of tonic currents by allopregnanolone in basolateral amygdala (BLA) interneurons, in contrast to diazepam. **(A)** Left panel. A representative image illustrating the mouse BLA slice and the recording interneuron with fast-spiking activity. Right panel. Representative traces indicated that allopregnanolone (Allo, 100 nM) potentiated tonic currents, as shown by inward currents (upper trace), while diazepam (Dia, 100 nM) had no effect on the BLA interneuron in wild-type mice with the SDS model (middle trace). In the GABA_A_ receptor δ-subunit knockout mouse (δko mouse) with the SDS model, allopregnanolone (Allo, 100 nM) did not potentiate tonic currents in the BLA interneuron (bottom trace). Application of bicuculine (10 μM) abolished inhibitory postsynaptic currents. **(B)** Allo (depicted in red) significantly potentiated tonic currents. ^*^p = 0.0028, *n* = 11 slices from wild-type mice with the SDS model, paired *t-*test. Dia (depicted in blue) did not significantly increase tonic currents. *n* = 11 slices from wild-type mice with the SDS model. Allo (depicted in dark blue) had no effect on tonic currents. *n* = 6 slices from δko mice with the SDS model. **(C)** Left panel. Summary of the significant increase in the decay of inhibitory postsynaptic currents (IPSCs) by allopregnanolone (100 nM) in BLA interneurons from wild-type mice with the SDS model. ^**^p = 0.0067, *n* = 11, unpaired *t*-test. Right panel. Summary of the significant increase of the amplitude of IPSCs by allopregnanolone (100 nM) in BLA interneurons from wild-type mice with the SDS model. ^**^p = 0.0347, *n* = 11, unpaired *t*-test. **(D)** The same as **(C)**, but for diazepam (100 nM). ^*^p = 0.0251 for the decay, ^*^p = 0.0044 for the amplitude, *n* = 11, unpaired *t*-test.

Whole-cell voltage-clamp recordings were made from visually identified interneurons in the BLA area using an upright microscope with infrared differential interference contrast optics. The recorded neurons exhibited sustained fast spiking activity following the current injection (Figure 2A), consistent with a previous study (Park et al., 2007; Perumal and Sah, 2022). Patch electrodes (2.5–3.0 μm tip diameter) were pulled from borosilicate glass capillaries and had a resistance of 3–5 MΩ when filled with an internal solution consisting of 135 mM KCl, 10 mM HEPES, 1.1 mM EGTA, 2 mM MgCl_2_, 3 mM Mg-ATP, and 0.3 mM Li–GTP, pH 7.3, adjusted with KOH. Membrane voltage was recorded with a patch clamp system (EPC-10, HEKA, Darmstadt, Germany) and PowerLab (ADInstruments, Dunedin, New Zealand), low-pass filtered at 4 kHz, and digitized at 40 kHz for computer analysis using Pulse software (HEKA) and LabChart software (ADInstruments). All experiments were performed at 30–32°C. To block excitatory postsynaptic currents, 10 μM CNQX and 50 μM D-AP5 were added to standard artificial CSF. To unmask the contribution of tonic current, 10 μM bicuculline was perfused at the end of each recording.

### Data analysis

The magnitude of the tonic current was calculated as previously described (Antonoudiou et al., 2022). The mean current was measured during 10 ms epochs collected every 100 ms throughout the recording. A histogram of the holding currents (30 s before the application of bicuculline) and in the presence of bicuculline (30 s after bicuculline blockade) were fit with a Gaussian, and the difference in the mean of the fitted curve was defined as posttonic currents. Pretonic currents were measured by calculating the bicuculline-induced shift in holding currents from baseline (prior to the application of allopregnanolone or diazepam). Regarding the analysis of spontaneous inhibitory postsynaptic currents (sIPSCs), the data of amplitude, frequency, and decay were analyzed using easy electrophysiology (Easy Electrophysiology Ltd., London, United Kingdom). The amplitude, frequency, and decay of sIPSCs for 30 s during pre- and post-treatment of allopregnanolone and diazepam were calculated by averaging the values of individual sIPSCs, which were detected based on criteria. Decay was measured as τ by fitting individual sIPSCs with exponential functions. Data are expressed as pre- and post-treatment parameters. Statistical analysis was conducted using a paired *t*-test for pre- and post-treatment comparisons. For statistical analysis for comparisons between naïve mice and SDS models, an unpaired *t*-test was performed using pre-treatment parameters for each group. Cells were excluded from the analysis if series resistance or whole-cell capacitance changed >30% during the course of the recording.

### *In vivo* electrophysiological assessment

#### Surgery

Surgery was performed to implant electrodes for recording LFPs, EEGs, and electromyography (EMG) signals from mice. The detailed surgical procedures have been previously described in Okada et al., 2017; Sasaki et al., 2017; Shikano et al., 2018, and Yawata et al., 2023. Briefly, LFP electrode devices consisting of a core body and a custom-made electrical interface board accommodating 6–12 LFP channels, 2 EMG channels, and 1 ground/reference channel were assembled for LFP recordings before surgery. For the surgery, mice were anesthetized with 1–2.5% isoflurane gas. After anesthesia, a midline incision was made from the area between the eyes to the incised neck area, and two stainless-steel EMG electrodes with a tip diameter of 147 μm (AS633; Cooner Wire Company) in which the PTFE coating at the tip (~5.0 mm) was peeled off were sutured to the dorsal neck muscles. Circular craniotomies with a diameter of ~1 mm were made using a high-speed drill above the mPFC (1.9 mm anterior and 0.2 mm right to the bregma), BLA (1.6 mm posterior and 3.0 mm right to the bregma), and cerebellum (5.8 mm posterior ±1.0 mm lateral to the bregma) for the ground/reference. The dura was surgically removed. The tips of the BLA electrodes were inserted 3.85 mm from the brain surface. Stainless-steel screws were implanted on the surface of the cerebellum (5.8 mm posterior and 1.0 mm right/left to the bregma) as ground/reference electrodes. All the wires and the electrode assembly were secured to the skull using dental cement.

Socket pins (ME-3-1, MAC8 Co., Ltd.) were used for the EEG electrodes. An EMG electrode to use in conjunction with EEG recordings was created by soldering the socket pin to a wire (0.26ETFE2X7, Junkosya, Ibaraki, Japan). Electrode implantation surgery was similar to EEG and LFP recording. However, electrodes implanted above the frontal cortex (1.5 mm anterior and ± 0.2 mm lateral to the bregma), parietal cortex (2.0 mm posterior and ± 2.5 mm lateral to the bregma), and cerebellum (5.8 mm posterior ±1.0 mm lateral to the bregma) were used for the ground/reference.

#### *In vivo* electrophysiological recording

Approximately 1 week after surgery, each mouse was connected to recording equipment to record *in vivo* LFP and EEG signals. Recordings were conducted within a copper-shielded room to remove 60-Hz hum noise. LFPs were sampled at 2 kHz using Cerebus (Blackrock Microsystems, Salt Lake City, Utah, United States), and EEG data were sampled at 400 Hz using the PowerLab system (ADInstruments, Dunedin, New Zealand). The LFP data were filtered between 0.1 and 500 Hz. Home-cage recordings were conducted for 60 min. After a baseline recording for 30 min, the mice were administered vehicle, diazepam (2 mg/kg), or allopregnanolone (20 mg/kg) intraperitoneally, and recording continued for an additional 30 min.

#### Histology

After all recordings and behavioral tests, mice implanted with LFP electrodes were euthanized with an overdose of isoflurane and perfused intracardially with PBS, followed by 4% paraformaldehyde in PBS. Brains were removed, postfixed overnight in 4% paraformaldehyde, and equilibrated in 30% sucrose in PBS overnight. Frozen coronal sections (50 μm) were cut using a cryostat (NX50, PHC Corporation) and mounted with a DAPI-containing mounting medium (VECTASHIELD Vibrance Antifade Mounting Medium, Funakoshi). Fluorescence images were captured using an all-in-one microscope (BZ-X710; Keyence, Osaka, Japan). LFP recordings were excluded from the data analysis unless the electrode was in the mPFC and BLA.

#### Data analysis

To analyze LFP and EEG signals within the physiological range, periods in which the LFPs for 1-s bins exceeded 2 mV were excluded from the analysis. In home-cage recordings, we included data from mice with excluded time periods comprising less than 10% of the total. Moreover, to identify periods containing noise from mouse movement, EMG was used as previously described (Konno et al., 2022). Briefly, a high-pass filter with a cutoff frequency of 100 Hz was applied to the EMG signal, and the root mean square was calculated for 1-s bins (rmsEMG). The mean plus one standard deviation of rmsEMG was used as a threshold, and periods in which the rmsEMG exceeded the threshold were excluded from the analysis. The missing values during the excluded periods were filled using linear interpolation. The Fourier transform was applied to LFP and EEG signals, and the intensity of each frequency was calculated for every 1-s bin. The definitions of each frequency band were based on previous studies (Antonoudiou et al., 2022) and set as follows: theta wave (6–12 Hz) and beta wave (15–30 Hz). LFP and EEG power were computed using the NeuroExplorer (Plexon Inc., Dallas, Texas, United States) and the LabChart Reader (ADInstruments), respectively. The power values were normalized for each time point across the total power. The average power from 0 to 15 min before drug administration was defined as the preadministration period, and the average power from 15 to 30 min after administration was defined as the postadministration period. Differences between groups were analyzed for statistical significance with the paired *t*-test for two groups in LFP recordings from wild-type mice with the SDS model. Regarding LFP recordings from Gabrd^−/−^ mice with the SDS model and EEG recordings from naïve mice and SDS mice, unpaired *t-*tests were used for two groups. In the EEG analysis with naïve mice and SDS mice, Tukey’s test was used for multiple groups. A *p*-value of <0.05 was taken to indicate statistical significance. All data are expressed as the means ± SEMs.

## Results

### Rapid onset of the antidepressant-like effects of allopregnanolone, in contrast to diazepam

To investigate the differences in effects on depression-like behavior, we conducted a direct comparison study with the neuroactive steroid allopregnanolone and the benzodiazepine diazepam by using SIT in SDS model mice. This model features a variety of symptoms and pathologies of MDD and allows testing of the antidepressant-like effects of drugs (Petković and Chaudhury, 2022). Consistently, mice showed robust decreased social behavior time as a depressive symptom due to stress exposure compared to non-stress mice or before stress (Supplementary Figure 1). To conduct direct comparison studies, we utilized frontal beta oscillation with EEGs, a marker of GABA_A_ receptor potentiation. The dose of diazepam at 2 mg/kg (i.p.) employed in this study was established based on a notable enhancement in frontal beta activity (Supplementary Figure 2) and the manifestation of the anxiolytic-like effects (Supplementary Figure 4) at a non-sedative dosage (Supplementary Figures 3, 4). These results indicate GABA_A_ receptor potentiation subsequent to brain exposure (Friedman et al., 1992; Bhatt et al., 2013; Shahnouri et al., 2016; Antonoudiou et al., 2022). Similarly, the dose of allopregnanolone at 20 mg/kg (i.p.) in this study was non-sedating (Supplementary Figure 3) and determined by the observed increase in beta activity (Supplementary Figure 2). This setting dose of each drug is based on previous reports (Brot et al., 1997; Doukkali et al., 2016; Shahnouri et al., 2016; Holmberg et al., 2018; Dornellas et al., 2020; Antonoudiou et al., 2022; Choudhary et al., 2022). Before a direct comparison study between these agents, we first investigated whether the SIT was suitable for evaluating antidepressant-like effects by administering escitalopram, a selective serotonin reuptake inhibitor (SSRI) antidepressant, to mice to examine whether it improved their social interaction. Based on a previous study (Burstein et al., 2017), escitalopram at a dose of 10 mg/kg (p.o.) for 4 weeks increased social interaction, confirming that the SIT can be used to evaluate the antidepressant-like effects of SDS model mice (Figure 1B). This result also demonstrated the delayed onset of the antidepressant-like effects of escitalopram. With this behavioral test, we assessed the antidepressant-like effects of allopregnanolone and diazepam. Allopregnanolone at a dose of 20 mg/kg (i.p.) induced a rapid onset of antidepressant-like effects in the SIT of SDS mice (Figure 1C). In contrast, diazepam at a dose of 2 mg/kg (i.p.) had no effect at each time point from day 1 to day 7 (Figure 1C). Since escitalopram elicited a delayed onset of antidepressant-like effects in the SIT (Figure 1B), and diazepam, at a dose of anxiolytic-like effects, had no effects in the SIT test (Figure 1C), the effects in SIT could reflect efficacy on social interaction impairment or a lack of motivation to interact with novel conspecifics as dysregulated essential behaviors in rodents after social defeat stress (Petković and Chaudhury, 2022). With the TST, allopregnanolone (20 mg/kg, i.p.) but not diazepam (2 mg/kg, i.p.) elicited antidepressant-like effects (Supplementary Figure 5). The effects of the TST could reflect efficacy on behavioral despair and helplessness under unbearable environmental stressful conditions (Hao et al., 2019). These results demonstrated that allopregnanolone elicited antidepressant-like effects, while diazepam had no effect on depressive-like symptoms. Regarding the molecular mechanism underlying this different antidepressant-like effect, previous studies have reported that neuroactive steroids act on both extra and synaptic GABA_A_ receptors, while benzodiazepines primarily act on synaptic GABA_A_ receptors (Carver and Reddy, 2013; Althaus et al., 2020). Therefore, we investigated whether the potentiation of δ-subunit-containing GABA_A_ receptors might contribute to the *in vivo* efficacy of allopregnanolone by using Gabrd^−/−^ mice with SDS. The antidepressant-like effect of allopregnanolone was abolished in Gabrd^−/−^ mice (Figure 1D). This result demonstrated that δ-subunit-containing GABA_A_ receptors are involved in the rapid antidepressant-like effect of allopregnanolone.

### Increase in tonic inhibition in BLA interneurons with allopregnanolone, in contrast to diazepam

Signaling through δ-subunit-containing GABA_A_ receptors strongly influences network activity due to the role of tonic GABA currents in controlling the excitability of inhibitory interneurons (Vida et al., 2006; Lee and Maguire, 2014; Pavlov et al., 2014). A previous study demonstrated that allopregnanolone-induced potentiation of δ-subunit-containing GABA_A_ receptors in BLA interneurons could be related to its antidepressant-like effect (Antonoudiou et al., 2022; Luscher et al., 2023; Walton et al., 2023). This potentiation of δ-subunit-containing GABA_A_ receptors could be measured as tonic GABA currents in neurons (Vida et al., 2006; Lee and Maguire, 2014; Pavlov et al., 2014). Therefore, in the present study, we first performed whole-cell patch-clamp recording from BLA interneurons, which were visually identified and exhibited fast spiking activity by current injections, in slices prepared from SDS mice with depressive symptoms (Figure 2A). Allopregnanolone significantly potentiated tonic currents compared with diazepam in BLA slices from wild-type mice with the SDS model (Figure 2B, *P* = 0.0028, *n* = 11 slices, paired *t-*test). The augmentation of tonic currents induced by allopregnanolone was not observed in Gabrd−/− mice with the SDS model, thereby confirming the involvement of δ-subunit-containing GABA_A_ receptors in the currents (Figure 2B, *N* = 6 slices). Regarding phasic currents, both allopregnanolone and diazepam exhibited an augmentation of the amplitude and decay of IPSCs in BLA slices from wild-type mice with the SDS model (Figures 2C,D). The result obtained from this experiment confirms our understanding of the compound concentrations required for the potentiation of synaptic GABA_A_ receptors. Of greater significance, there was a notable reduction in the frequency of IPSCs in BLA interneurons from SDS mice during the pre-treatment period when compared to those from naïve mice (Supplementary Figure 6). This suggests that GABA release from presynaptic terminals might be diminished in a depressive state. Regarding the amplitude and decay of sIPSCs and tonic currents during the pre-treatment period, there were no significant changes between SDS and naïve mice (Supplementary Figure 6). Based on these findings, in conjunction with behavior outcomes, it can be inferred that the enhancement of extrasynaptic GABA_A_ receptors in BLA interneurons plays a role in the antidepressant-like effects of allopregnanolone, which diverge from the effects of diazepam.

### Distinct effect of allopregnanolone on resting BLA theta activity, in contrast to diazepam

**Figure 3:**
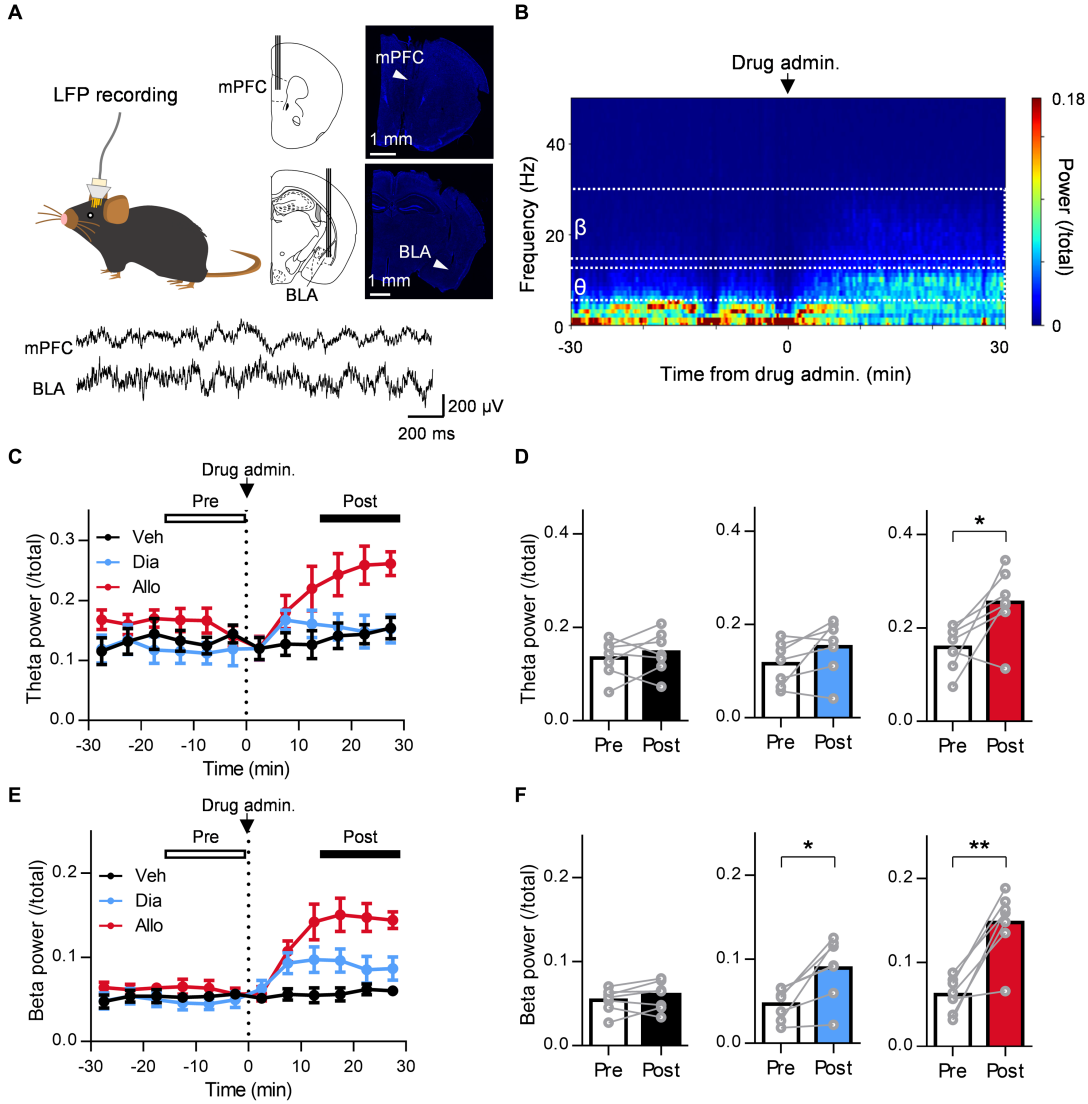
A distinct effect of allopregnanolone, in contrast to diazepam, on resting theta activity in the BLA. **(A)** Local field potentials (LFPs) were recorded from the medial frontal cortex (mPFC) and BLA of SDS mice. The arrowhead in the macrographs indicates the location of the electrode tip. The nuclei were counterstained with DAPI. The bottom panel demonstrates a representative trace of LFPs in the mPFC and BLA. **(B)** Representative power spectrogram of BLA LFPs before and after allopregnanolone administration. The LFP power was normalized for each time point across the total power and plotted on a pseudocolor scale. **(C)** Time course of BLA theta oscillation (6–12 Hz) power before and after administration of vehicle (Veh, depicted in black), diazepam (Dia, 2 mg/kg, depicted in blue), and allopregnanolone (Allo, 20 mg/kg, depicted in red). The dashed line indicates the timing of drug administration. Power values were normalized by total power and shown as ratios. The average power from 0 to 15 min before drug administration was defined as the preadministration period, and the average from 15 to 30 min after administration was defined as the postadministration period. Data are represented as the mean ± SEM of seven mice. **(D)** BLA theta power during the preadministration and postadministration periods with Veh (black), Dia (blue), and Allo (red). ^*^p = 0.026, *n* = 7 mice, paired *t-*test. Power values were normalized by total power and shown as ratios. **(E,F)** The same as **(C,D)** but for the beta oscillation (15–30 Hz) power. ^*^p = 0.011, ^**^p = 0.0014, *n* = 7 mice, paired *t-*test.

**Figure 4:**
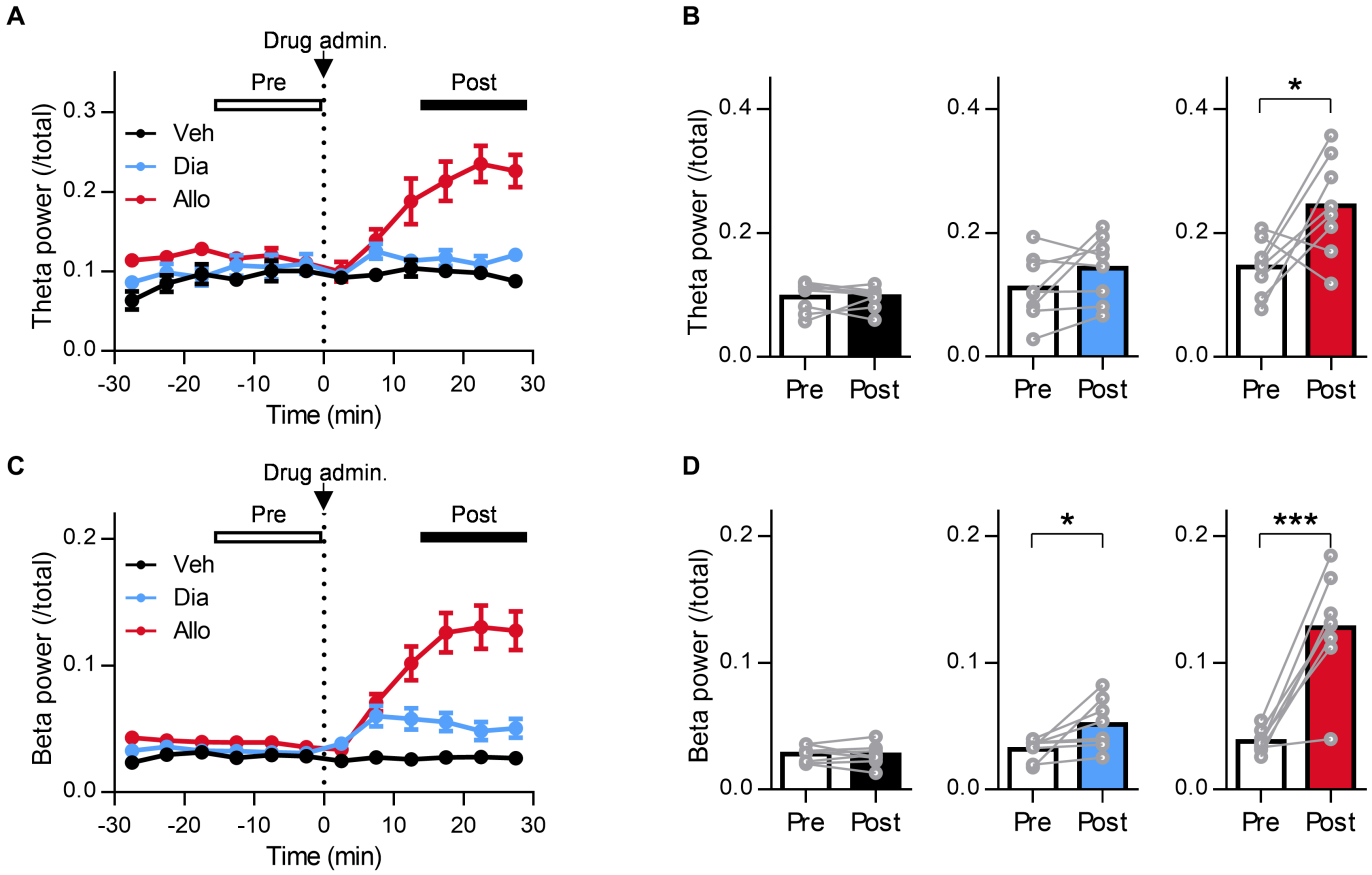
A distinct effect of allopregnanolone, in contrast to diazepam, on resting theta activity in the mPFC. **(A)** Time course of mPFC theta power before and after administration of vehicle (Veh, depicted in black), diazepam (Dia, depicted in blue), and allopregnanolone (Allo, depicted in red). The dashed line indicates the timing of drug administration. Data are represented as the mean ± SEM of eight mice. **(B)** mPFC theta power during the preadministration and postadministration periods for Veh (black), Dia (blue), and Allo (red). ^*^p = 0.03, *n* = 8 mice, paired *t-*test. Power values were normalized by total power and are shown as ratios. **(C,D)** The same as **(A,B)** but for beta power. ^*^p = 0.034, ^***^p = 0.0004, *n* = 8 mice, paired *t-*test.

A previous study reported that orchestrating the neural network centered around the amygdala is important for the mechanism of action of the antidepressant effects of neuroactive steroids (Antonoudiou et al., 2022). In particular, theta oscillations (6–12 Hz) in the BLA are essential for them as optogenetically induced theta oscillations in the BLA reduce immobility time in the TST (Antonoudiou et al., 2022). Moreover, previous studies have reported that theta oscillation in the mPFC is also involved in the regulation of depression (Kuga et al., 2022). Thus, to compare the effect of the neuroactive steroid allopregnanolone and benzodiazepine diazepam on the oscillation of these brain regions in SDS mice, we simultaneously recorded LFPs from the BLA and mPFC (Figure 3A). We recorded LFPs of SDS mice in their home cages for 30 min as a baseline, followed by the administration of vehicle, diazepam, and allopregnanolone, and continued recording for another 30 min (Figure 3B). According to a previous study (Antonoudiou et al., 2022), we focused on theta oscillations as the activity related to antidepressant effects and beta oscillations (15–30 Hz) as a marker of functional activation of the GABA_A_ receptor. The BLA theta power significantly increased after the administration of allopregnanolone (Figures 3C,D; *P* = 0.026, *n* = 7 mice, paired *t-*test) but was not changed after the administration of vehicle or diazepam. The BLA beta power increased after the administration of diazepam and allopregnanolone (Figures 3E,F; *P* = 0.011 and 0.0014, respectively, *n* = 7 mice, paired *t-*test) but was not changed in the vehicle condition. Similarly, we analyzed mPFC theta and beta oscillations. The mPFC theta power significantly increased after the administration of allopregnanolone (Figures 4A,B; *P* = 0.034, *n* = 8 mice, paired *t-*test), while theta power was not changed after the administration of vehicle or diazepam. The PFC beta power increased after the administration of diazepam and allopregnanolone (Figures 4C,D; *P* = 0.034 and 0.0004, respectively, *n* = 8 mice, paired *t-*test) but was not changed in the vehicle condition. These results demonstrate that allopregnanolone increases both theta and beta oscillations in the BLA and mPFC, while diazepam increases only beta activity. Moreover, when combined with our behavioral data in the SIT and TST, theta activity might be associated with the antidepressant effects of allopregnanolone.

### Involvement of δ-subunit-containing GABA_A_ receptors in the increase in resting theta activity in the BLA and mPFC due to allopregnanolone but not in beta activity

**Figure 5:**
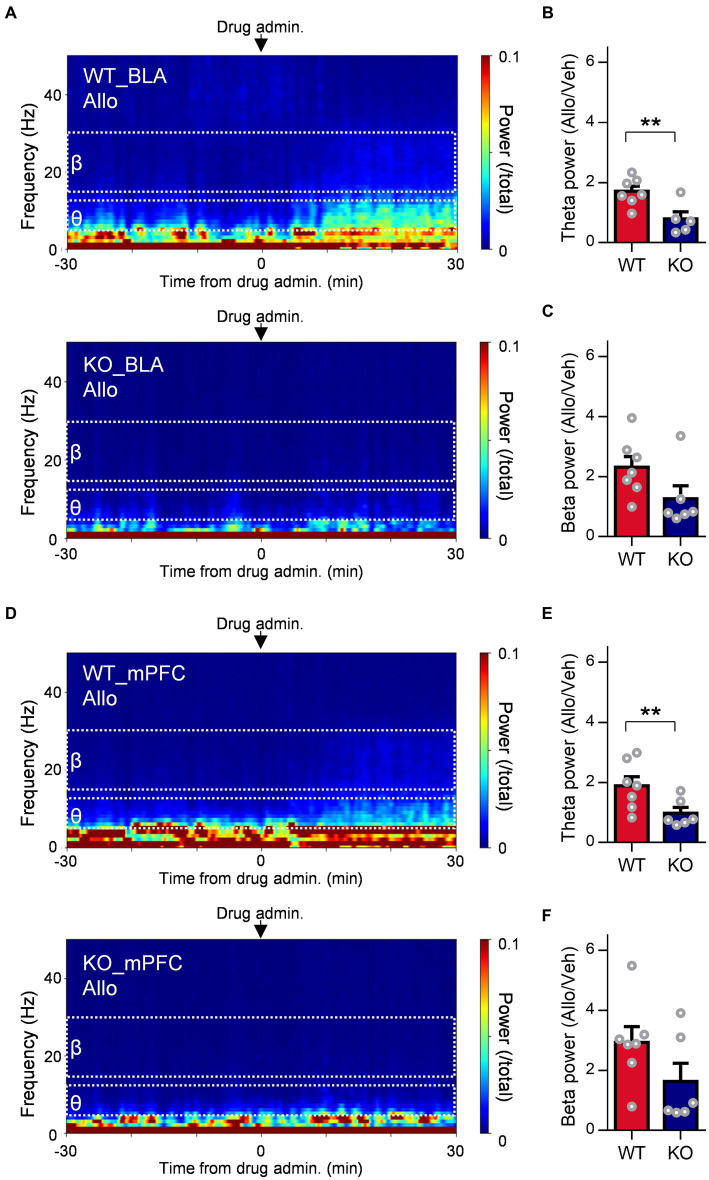
Involvement of extrasynaptic GABA_A_ receptors in the increase in resting theta activity in the BLA due to allopregnanolone. **(A)** Representative power spectrogram of BLA LFPs before and after allopregnanolone (Allo, 20 mg/kg) administration in a WT mouse (top) and a GABA_A_ receptor δ-subunit KO mouse (bottom). The LFP power was normalized for each time point across the total power and plotted on a pseudocolor scale. **(B)** The ratio of BLA theta power after the administration of Allo to that after the administration of vehicle (Veh) in WT (red) and KO (dark blue) mice. ^**^p = 0.0097, *n*_WT_ = 8 mice, *n*_KO_ = 5 mice, unpaired *t-*test. **(C)** The same as **(B)**, but for the ratio of BLA beta power. *p* = 0.088, *n*_WT_ = 8 mice, *n*_KO_ = 5 mice, unpaired *t-*test. **(D)** The same as **(A)** but for the mPFC LFPs. **(E)** The same as **(B)** but for mPFC theta power. ^*^p = 0.031, *n*_WT_ = 9 mice, *n*_KO_ = 6 mice, unpaired *t-*test. **(F)** The same as **(C)** but for mPFC beta power. *p* = 0.13, *n*_WT_ = 9 mice, *n*_KO_ = 6 mice, unpaired *t-*test.

To evaluate whether δ-subunit-containing GABA_A_ receptors are involved in oscillation due to allopregnanolone as well as its antidepressant effect, we assessed the effects of allopregnanolone on BLA and mPFC activity in Gabrd^−/−^ mice with SDS. The increase in theta activity in the BLA and mPFC due to allopregnanolone was significantly attenuated in Gabrd^−/−^ mice in the SDS model compared with wild-type mice in the SDS model (Figures 5A,B; *P* = 0.0097, *n*_WT_ = 8 mice, *n*_KO_ = 5 mice, unpaired *t-*test). Beta activity was also attenuated but not significantly (Figures 5A,C; *P* = 0.088, *n*_WT_ = 8 mice, *n*_KO_ = 5 mice, unpaired *t-*test). Similar results were obtained in mPFC theta (Figures 5D,E; *P* = 0.031, *n*_WT_ = 9 mice, *n*_KO_ = 6 mice, unpaired *t-*test) and beta activity (Figures 5D,F; *P* = 0.013, *n*_WT_ = 9 mice, *n*_KO_ = 6 mice, unpaired *t-*test). Thus, theta activity in the BLA and mPFC mediated by δ-subunit-containing GABA_A_ receptors is essentially involved in the antidepressant effects of allopregnanolone.

### Distinct effect of allopregnanolone on frontal theta and beta activity, in contrast to diazepam

**Figure 6:**
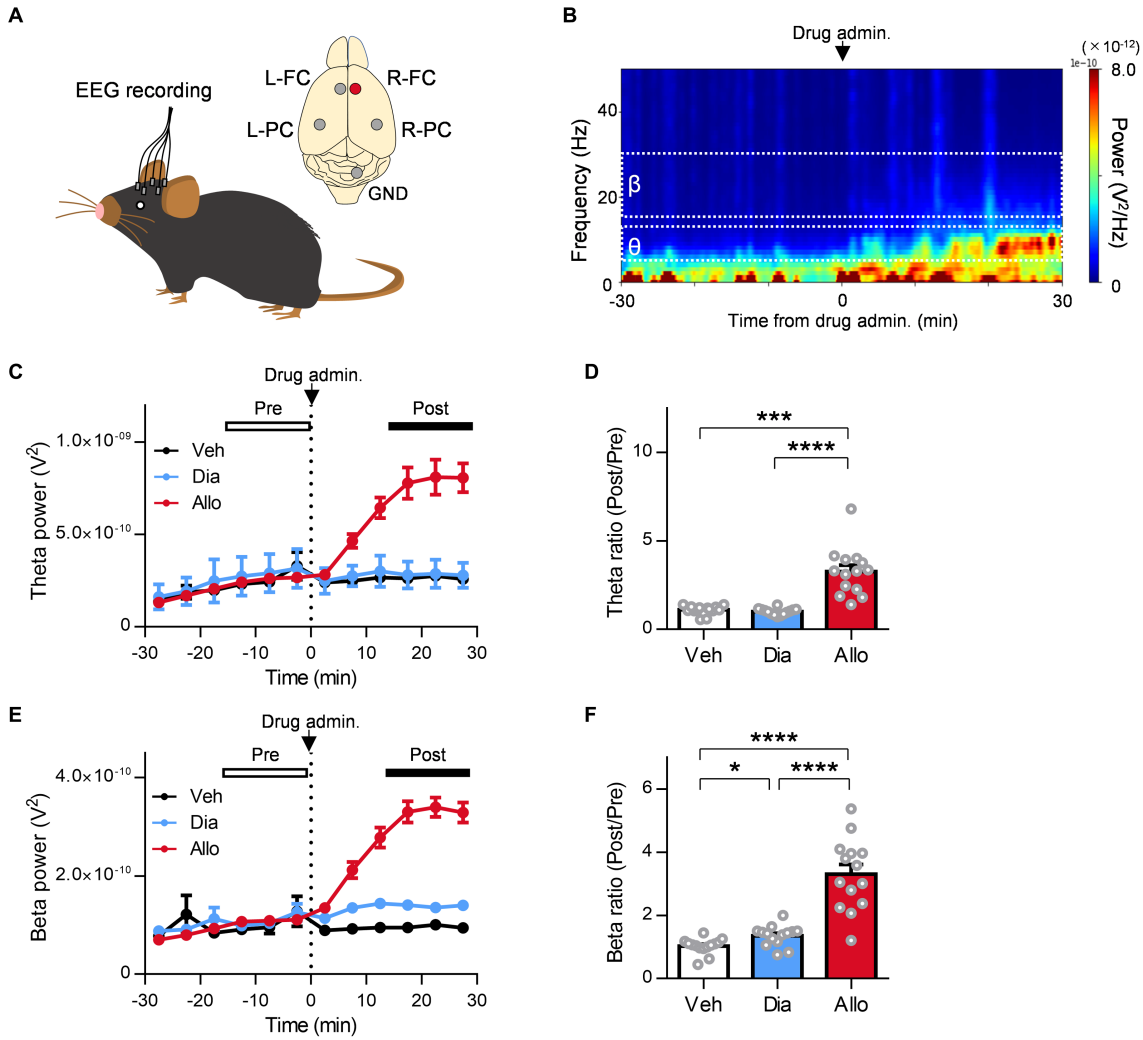
Distinct effect of allopregnanolone compared with diazepam on resting frontal EEG theta activity in SDS model mice. **(A)** Electroencephalograms (EEGs) were recorded from the bilateral frontal cortex (FC) and parietal cortex (PC) of naïve and SDS model mice. The EEGs of the right FC (R-FC) were used for the analysis. GND, ground electrode. **(B)** Representative power spectrogram of R-FC EEGs before and after allopregnanolone administration. **(C)** Time course of R-FC theta power before and after the administration of vehicle (Veh, depicted in black), diazepam (Dia, depicted in blue), and allopregnanolone (Allo, depicted in red). The dashed line indicates the timing of drug administration. Data are represented as the mean ± SEM of 14 mice. **(D)** The ratio of R-FC theta power during the postadministration to the preadministration for Veh (black), Dia (blue), and Allo (red). ^***^p = 0.0003 and ^****^p < 0.0001 for vehicle versus allopregnanolone and diazepam versus allopregnanolone, respectively, *n* = 14 mice, Tukey’s test. **(E,F)** The same as **(C,D)**, but for beta power. ^*^p = 0.040, ^****^p < 0.0001, *n* = 14 mice, Tukey’s est.

A previous study reported that neuroactive steroids such as zuranolone increase the theta activity of the frontal cortex in healthy volunteers based on EEG recordings (Antonoudiou et al., 2022), while benzodiazepines such as diazepam do not (Friedman et al., 1992). Regarding the increase in frontal beta activity, reflecting the functional activation of GABA_A_ receptors in the brain, both neuroactive steroids and benzodiazepine have effects in healthy volunteers (Friedman et al., 1992; van Broekhoven et al., 2007; Antonoudiou et al., 2022). However, no clinical evidence has been reported as direct comparison data between them in the same protocol. Therefore, in reference to these clinical studies, we evaluated the effects of allopregnanolone and diazepam on frontal theta and beta activity in EEG recordings from naïve mice and the SDS model (Figures 6A,B). In SDS model mice, allopregnanolone significantly increased frontal theta activity (Figures 6C,D; *P* = 0.0003, *p* < 0.0001 for vehicle versus allopregnanolone and diazepam versus allopregnanolone, respectively, *n* = 14 mice, Tukey’s test), while diazepam did not. Regarding frontal beta activity, both allopregnanolone and diazepam significantly increased it (Figures 6E,F; *P* = 0.040, *p* < 0.0001, 0.0001 for vehicle versus diazepam, diazepam versus allopregnanolone and vehicle versus allopregnanolone, respectively, *n* = 14 mice, Tukey’s test). A similar result was obtained in naïve mice (Supplementary Figure 2). Thus, we have demonstrated that there is a difference in theta and beta activity in the frontal cortex between allopregnanolone and diazepam in a direct comparison study with EEG recordings from naïve and SDS model mice.

### Decrease in frontal beta and theta activities in the depressive state

**Figure 7:**
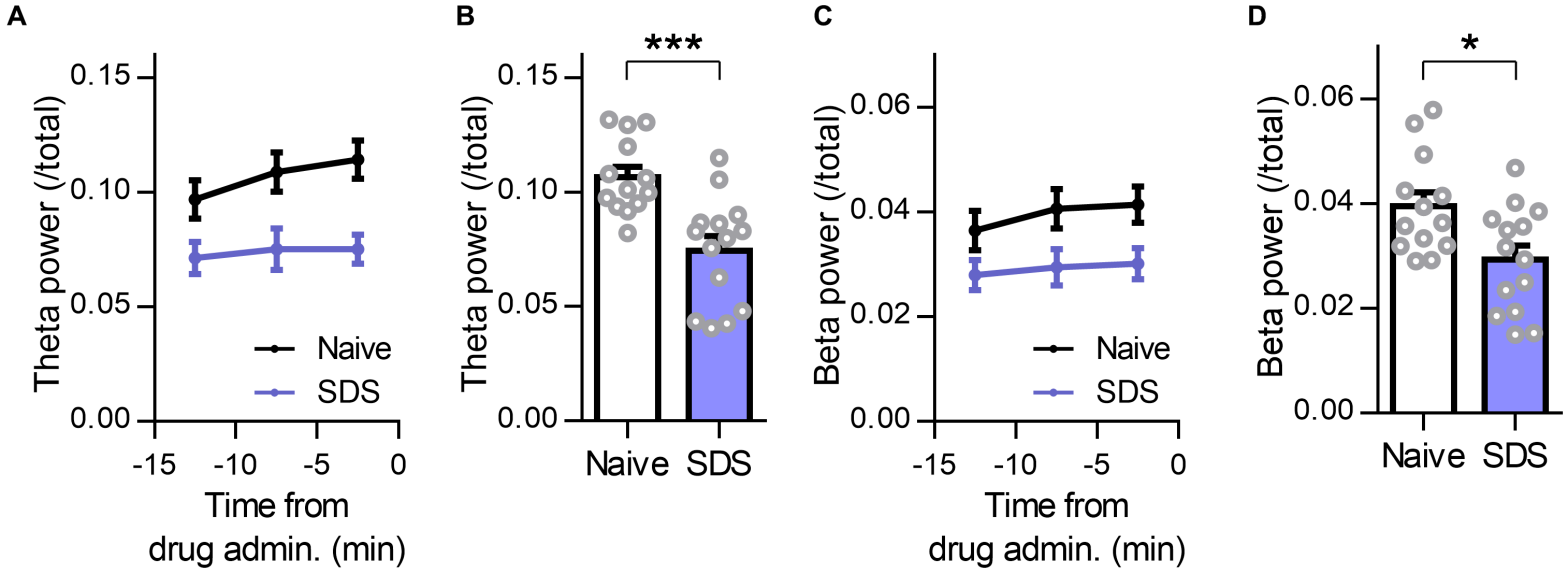
Decrease in frontal beta and theta activities in the depressive state. **(A)** Time course of the R-FC theta power of naïve (black) and SDS (purple) mice before administration of the drug. The average preadministration period of all drugs (vehicle, diazepam, and allopregnanolone) was used. The data are represented as the mean ± SEM of 14 mice each. **(B)** The R-FC theta power of naïve and SDS mice during the preadministration period of drugs. ^***^p = 0.0004, *n* = 14 mice each, unpaired *t-*test. **(C,D)** The same as **(A, B)**, but for beta power. ^*^p = 0.013, *n* naïve = 13 mice, *n* _SDS_ = 14 mice, unpaired *t-*test.

Finally, we evaluated changes in theta and beta activity in the frontal cortex of SDS model mice. Several previous studies reported that dysfunction of GABAergic inhibition, such as the amount of GABA in the frontal cortex and the expression of GABA_A_ receptors or their function, is reduced not only in animal models but also in depressed patients (Luscher et al., 2011). Furthermore, allopregnanolone increased frontal theta and beta activity in depressed SDS mice. Therefore, we hypothesized that theta activity and beta activity in the frontal cortex of SDS model mice were lower than those of naïve mice. We calculated the theta power and beta power during the preadministration period as baseline activity and compared them between naïve and SDS model mice (Figures 7A,C). As a result, both theta power and beta power in the frontal cortex of SDS mice were significantly lower than those of naïve mice (Figures 7B,D; *P* = 0.0004 and 0.0127 for theta power and beta power, respectively, *n* = 14 mice each, unpaired *t-*test). These results showed that baseline activity in the theta and beta bands decreases in the SDS model.

## Discussion

### Rapid onset of antidepressant-like effects of allopregnanolone, in contrast to diazepam

In the present study, allopregnanolone elicited antidepressant-like effects in the SIT and TST of the SDS model. In contrast, diazepam had no effect on depression-like behaviors. The dose of diazepam used in the present study was determined by its anxiolytic-like effects and increased beta activity of the frontal cortex without sedation, which is within a range of previous studies (Bhatt et al., 2013; Shahnouri et al., 2016; Antonoudiou et al., 2022). Similarly, the dosage of allopregnanolone was set to increase beta activity without sedation. The present result of the locomotor assessment and EEG confirms our understanding of the dose of compound required for the potentiation of GABA_A_ receptors and antidepressant or anxiolytic effects without sedation. In the SIT of the SDS model, we demonstrated that allopregnanolone elicited rapid-onset antidepressant-like effects. Diazepam, at a dose eliciting anxiolytic-like effects, had no effect on the SIT of SDS mice. Escitalopram for 4 weeks elicited significant antidepressant-like effects. Based on these results, the present study demonstrated rapid onset effects of allopregnanolone on depression-like behavior in the SIT of the SDS model, in contrast to diazepam and escitalopram. This corresponds to clinical evidence of the efficacy profile of these drugs on depressive symptoms in MDD patients (Rush et al., 2006; Lim et al., 2020; Edinoff et al., 2021). More importantly, this antidepressant-like effect of allopregnanolone in SIT was abolished in δΚΟ mice. These data demonstrated that δ-subunit-containing GABA_A_ receptors are critical for the antidepressant-like effects of allopregnanolone. Both diazepam and allopregnanolone are positive allosteric modulators of GABA_A_ receptors, exhibiting relatively little α-subunit selectivity. Diazepam acts on the benzodiazepine site of α1, 2, 3, and α5-containing GABA_A_ receptors (Carver and Reddy, 2013; Nuss, 2015). Allopregnanolone is a non-selective neuroactive steroid acting on α1, 2, 3, 4, 5, and α6-containing GABA_A_ receptors (Sigel, 2002; Hosie et al., 2006; Carver and Reddy, 2013; Nuss, 2015; Alvarez and Pecci, 2018). In combination with genetically modified animals, the relationship between each α subunit and behavior has been shown, particularly regarding the effects of benzodiazepines; for example, the α1 subunit for sedation and the α2 subunit for anxiolysis (Engin et al., 2018). In the present study, the relief of depressive symptoms by allopregnanolone is associated with δ-subunit-containing GABA_A_ receptors. Particularly, recent studies report that not only δ-subunit-containing GABA_A_ receptors but also other subtypes of GABA_A_ receptors such as the α5-subunit have been related to behaviors such as affective and cognitive function in the depression model (Piantadosi et al., 2016; Luscher et al., 2023). Thus, each subunit-selective behavioral profile and their associated neuronal activities remain to be clarified in future studies. Although potentiation of δ-subunit-containing GABA_A_ receptors by allopregnanolone may be critical for mediating the rapid antidepressant effects, it could not be excluded that phasic inhibition via synaptic GABA_A_ receptors by allopregnanolone could contribute to antidepressant effects. Future studies with the effects of DS2, which is a selective compound for δ-subunit-containing GABA_A_ receptors, are needed in the SDS model. Moreover, previous studies have shown that SSRIs elicit moderate and slow increases in allopregnanolone, associated with the initiation of their antidepressant effects (Romeo et al., 1998). Further studies of the effects of SSRIs, combined with δKO mice in the SDS model, are needed to clarify the involvement of δ-subunit-containing GABA_A_ receptors in the effects of SSRIs.

### Distinct neurophysiological mechanism of allopregnanolone acting on BLA oscillation, in contrast to diazepam

The amygdala is one of the abundant regions with a high concentration of neuroactive steroids (Bixo et al., 2018) and is involved in vigilance attention and learning biologically relevant signals such as negative emotional stimuli (Tzovara et al., 2019). Altered amounts of neuroactive steroids have been implicated in amygdala reactivity to negative emotional stimuli and stress vulnerability (Pisu et al., 2022). Dysregulation of neural network activity involving the amygdala is associated with depressive symptoms in patients with MDD (Drevets, 2000, Phillips et al., 2003). Recent studies demonstrated that neuroactive steroid treatment increased BLA theta activity in stress model mice and elicited antidepressant-like effects (Antonoudiou et al., 2022; Luscher et al., 2023; Walton et al., 2023). In addition, optogenetic stimulation of BLA interneurons at a theta frequency of 8 Hz caused antidepressant effects in a chronic unpredictable stress mouse model (Antonoudiou et al., 2022). The antidepressant-like effects of allopregnanolone were abolished in δKO mice (Antonoudiou et al., 2022). Thus, the activation of δ-subunit-containing GABA_A_ receptors in BLA interneurons is believed to contribute to the antidepressant-like effects of allopregnanolone by triggering an increase in BLA theta activity. In the present study, several differences between allopregnanolone and diazepam may contribute to antidepressant-like effects. Focusing on BLA interneurons, allopregnanolone potentiated tonic inhibition via potentiation of δ-subunit-containing GABA_A_ receptors. In addition, allopregnanolone but not diazepam increased theta activity in the BLA during the resting state in SDS mice. Increased theta activity was attenuated in δΚΟ mice. Allopregnanolone has been reported to act on δ-subunit-containing GABA_A_ receptors only in BLA interneurons but not in principal neurons (Antonoudiou et al., 2022). Thus, allopregnanolone-induced increases in the theta activity of the BLA through δ-subunit-containing GABA_A_ receptors in BLA interneurons may lead to a shift from fear to a normal state and reduced depression-like behaviors in the SDS model. With regard to beta activity within BLA, both allopregnanolone and diazepam exhibited enhancements, indicating that the augmentation of beta power resulted from the activation of synaptic GABA_A_ receptors in BLA interneurons. These results align with our observed potentiation of IPSC decay and amplitude in BLA interneurons by allopregnanolone and diazepam. In the present study, the allopregnanolone-induced increase in beta activity was slightly attenuated in δΚΟ mice but not significantly. This implies that δ-subunit-containing GABA_A_ receptors are also involved in both beta and theta activities in the BLA increased by allopregnanolone, which is consistent with a previous study (Antonoudiou et al., 2022). Considering the significant correlation between increased BLA theta activity and antidepressant effects from neuroactive steroids (Antonoudiou et al., 2022) and the increase in beta activity alone by diazepam without antidepressant-like effects, an increase in BLA theta activity via δ-subunit-containing GABA_A_ receptors may be important for the antidepressant-like effects of allopregnanolone. Of course, the possible involvement of beta activity in the antidepressant-like effects of allopregnanolone cannot be excluded. At the very least, the differences in the antidepressant-like effects of allopregnanolone and diazepam could be largely attributed to differences in the theta oscillation of the BLA. In addition to BLA activity, mPFC theta activity was increased by allopregnanolone but not by diazepam. This increase in theta activity in the mPFC was attenuated in δΚΟ mice. As for mPFC beta activity, both allopregnanolone and diazepam demonstrated an increase in activity. Consequently, not only theta activity in the BLA but also in the mPFC might be implicated in the antidepressant-like effects of allopregnanolone. Previous investigations have established that augmented theta activity in the mPFC and BLA plays a role in the regulation and discrimination of negative stimuli or emotions (Calhoon and Tye, 2015; Tovote et al., 2015). Specifically, it has been reported that BLA firing activity becomes synchronized with input from the mPFC, indicating that the BLA is selectively tuned to mPFC input, which potentially contributes to memory retrieval, fear responses, and extinction (Likhtik et al., 2014). It is worth noting that the function of the mPFC has also been associated with amygdala output (Senn et al., 2014). Indeed, allopregnanolone regulates the functional connectivity between the human frontal cortex and amygdala, which is involved in the relief of fear memory or cognition of negative stimuli (Sripada et al., 2014). In addition, an increase in theta activity in the mPFC and BLA in mice is also involved in approach behavior to communicate with other mice (Kuga et al., 2022). As an underlying neurological mechanism, modulation of interneuron activity not only in the BLA but also in the mPFC has been reported to contribute to antidepressant-like effects in preclinical models (Fogaça et al., 2021; Antonoudiou et al., 2022). This implies that allopregnanolone could affect not only emotional cognition but also motivational behavior. This is supported by previous reports that the amygdala and frontal cortex are involved in the regulation of motivational behavior via dopamine signaling pathways, which could be regulated by allopregnanolone (Rougé-Pont et al., 2002; Phillips et al., 2003; Holmberg et al., 2018). Thus, allopregnanolone, unlike diazepam, could ameliorate negative emotion and motivational affect, which is regulated by BLA-PFC theta activity.

### Distinct modulation of frontal theta and beta EEG activity by allopregnanolone, in contrast to diazepam, as a translational biomarker

Previous studies with EEGs have shown that neuroactive steroids such as zuranolone, but not benzodiazepines such as diazepam, increase frontal cortex theta activity in healthy volunteers (Romano-Torres et al., 2002; Saletu et al., 2006; Antonoudiou et al., 2022). Regarding frontal beta activity, a marker of central GABA_A_ receptor activation, both neuroactive steroids and benzodiazepines increased this activity (Friedman et al., 1992; Romano-Torres et al., 2002; Saletu et al., 2006; van Broekhoven et al., 2007; Antonoudiou et al., 2022). Interestingly, the increase in beta activity has been suggested to be more potent with allopregnanolone compared to diazepam, but no report has verified it in a head-to-head comparison under the same conditions. In the present study, we used an SDS model and naïve mice to demonstrate the difference in the effect of both agents in enhancing frontal theta activity by direct comparison verification. Furthermore, regarding beta activity, we observed that both allopregnanolone and diazepam increased this activity and that allopregnanolone exhibited a significant increase compared with diazepam. Thus, we have demonstrated distinct EEG profiles for allopregnanolone and diazepam, supported by previous studies (Visser et al., 2003; Antonoudiou et al., 2022; Hammond et al., 2022). This EEG result using the SDS model and naïve mice not only supports the results of the BLA and mPFC theta activity assessment in LFP recordings but also could be important as translational evidence forming a bridge from non-clinical evidence to human evidence. It is implied that frontal theta activity, similar to that in the BLA, is related to the mechanism of action of neuroactive steroids and may potentially contribute to the superior antidepressant-like effects of allopregnanolone. It has also been clinically reported that changes in frontal theta power could predict antidepressant efficacy in responders (Wade and Iosifescu, 2016; Wu et al., 2020). Since we did not investigate the causal relationship between theta activity and antidepressant effects, an additional study will be needed to examine the effects of theta activity on the downstream signals, such as stress responses, and its influence on antidepressant-like effects in depression model mice. Indeed, it has been reported that the optogenetically induced theta oscillations in the BLA have an antidepressant-like effect in the TST (Antonoudiou et al., 2022).

### The decrease in beta and theta activities in a depressive state and their increase in response to allopregnanolone, related to extrasynaptic GABA_A_ receptors

Decreased frontal beta and theta activities have been reported to correlate with the severity of depressive symptoms in patients with MDD (Saletu et al., 2010). Similarly, a decrease in frontal beta and theta activities was observed in SDS mice compared to non-stressed naïve mice, as evidenced by EEG recordings. Neuronal oscillation is potently regulated by GABAergic signaling through synaptic and extrasynaptic GABA_A_ receptors (Luscher et al., 2011). In particular, signaling through δ-subunit-containing GABA_A_ receptors strongly impacts network activity due to tonic inhibition regulating inhibitory interneuron excitability. Inhibitory interneurons are key regulators of rhythmic brain network activity (Vida et al., 2006; Mann and Paulsen, 2007). Recently, dysfunction of GABAergic inhibition has been reported in the pathology of MDD, such as a decrease in the concentration of GABA and/or allopregnanolone (Perlman et al., 2021), changes in the subunit expression of the GABA_A_ receptor (Luscher et al., 2011), and the excitability of GABAergic interneurons (Jie et al., 2018) in several brain areas including mPFC and BLA. Our present findings further support these reports as we observed a decrease in presynaptic GABA release in BLA slices from SDS mice. While the specific alterations in neuronal oscillation within the BLA during a depressive state remain to be fully elucidated, it is plausible that these mechanisms contribute to the observed decrease in frontal beta and theta activities in SDS mice. Importantly, our results demonstrated that allopregnanolone potentiated PFC-BLA beta and theta activities in SDS mice, and δ-subunit-containing GABA_A_ receptors are important mechanisms of action of allopregnanolone. In terms of molecular mechanisms, previous studies reported that δ-subunit-containing GABA_A_ receptors enable neurons to sense the low ambient GABA concentrations present in the extracellular space, leading to tonic inhibition and both cell and network behavior (Carver and Reddy, 2013; Feng and Forman, 2018). Additionally, neuroactive steroids, including allopregnanolone and zuranolone, can enhance the surface expression of GABA_A_ receptors, thereby amplifying GABA currents through multiple mechanisms (Abramian 2014; Modgil 2017). Thus, dysregulated neuronal network activity under a depressive state could be shifted to a normal state by treatment with neuroactive steroids. Additionally, several mood disorders, such as bipolar disorder, premenstrual dysphoric disorder, and postpartum depression, have dysregulated neuronal networks, along with changes in the concentration of allopregnanolone (Porcu et al., 2016; Karademir et al., 2023). Neuroactive steroids with the potentiation of synaptic and extrasynaptic GABA_A_ receptors may therefore present a potential therapeutic target for the treatment of major depressive disorder.

## Data Availability Statement

The raw data supporting the conclusions of this article will be made available by the authors, without undue reservation.

## Ethics Statement

The animal study was approved by All procedures were approved by the Animal Care and Use Committee of Shionogi Research Laboratories, Osaka, Japan. Electrophysiological assessments were performed according to the AAALAC International guidelines. The study was conducted in accordance with the local legislation and institutional requirements.

## Author contributions

KT: Conceptualization, Data curation, Investigation, Writing – original draft, Writing – review & editing. YY: Data curation, Formal analysis, Investigation, Methodology, Writing – original draft. RT: Data curation, Formal analysis, Investigation, Methodology, Writing – original draft, Writing – review & editing. HA: Data curation, Writing – review & editing. SS: Data curation, Writing – review & editing. TO: Project administration, Writing – review & editing. TT: Conceptualization, Project administration, Writing – review & editing. KO: Conceptualization, Investigation, Project administration, Writing – review & editing.

## Supplementary material

The Supplementary material for this article can be found online at:

https://www.frontiersin.org/articles/10.3389/fncel.2023.1274459/full#supplementary-material
