# Supplementary figures and images for: Distinct mechanisms of allopregnanolone and diazepam underlie neuronal oscillations and differential antidepressant effect
Source: Front Cell Neurosci. 2024 Jan 8;17:1274459. doi: 10.3389/fncel.2023.1274459 (PMC10800935; doi:10.3389/fncel.2023.1274459)

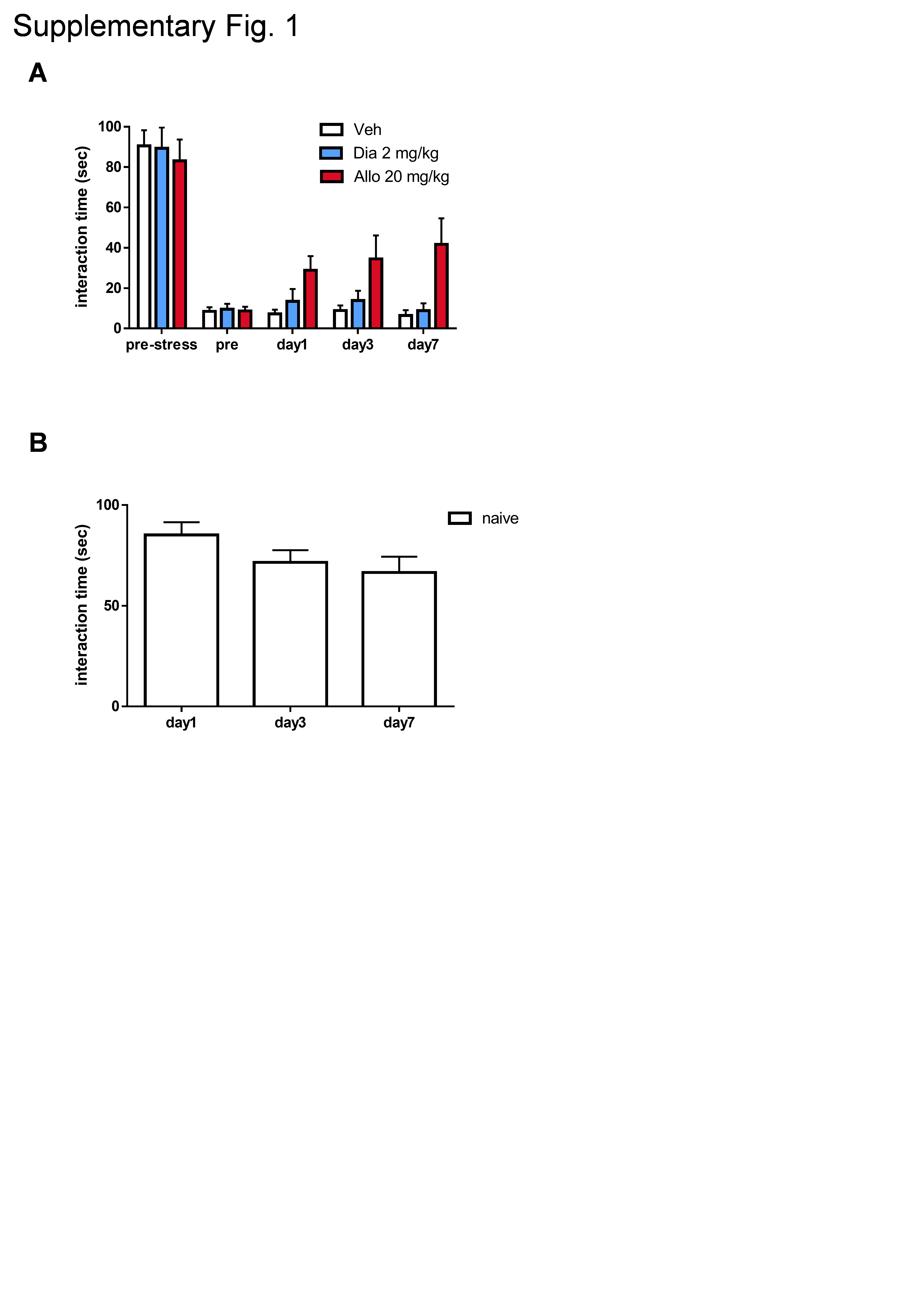

Supplement: Supplementary file 1 [file Image_1.tif]

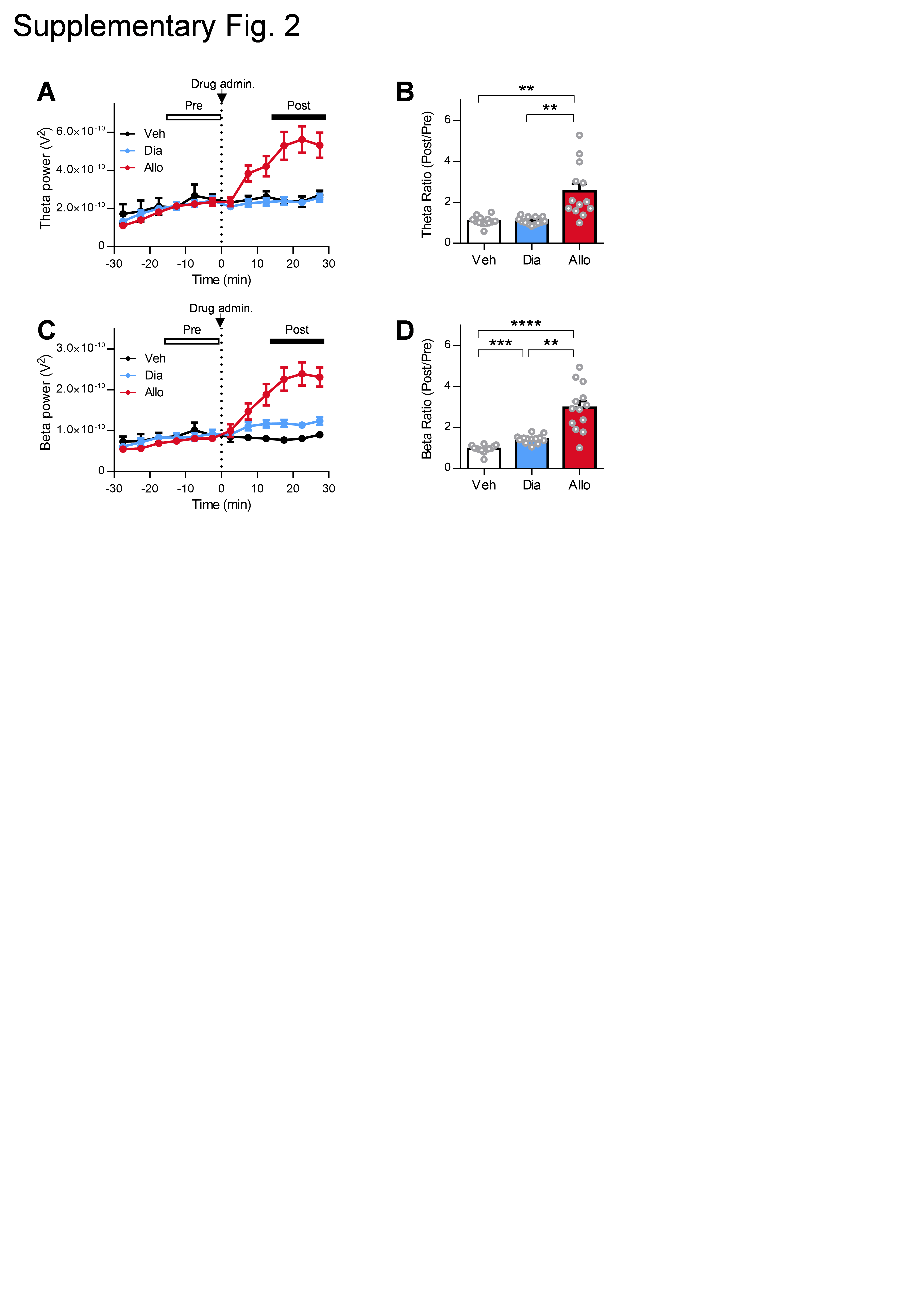

Supplement: Supplementary file 2 [file Image_2.tif]

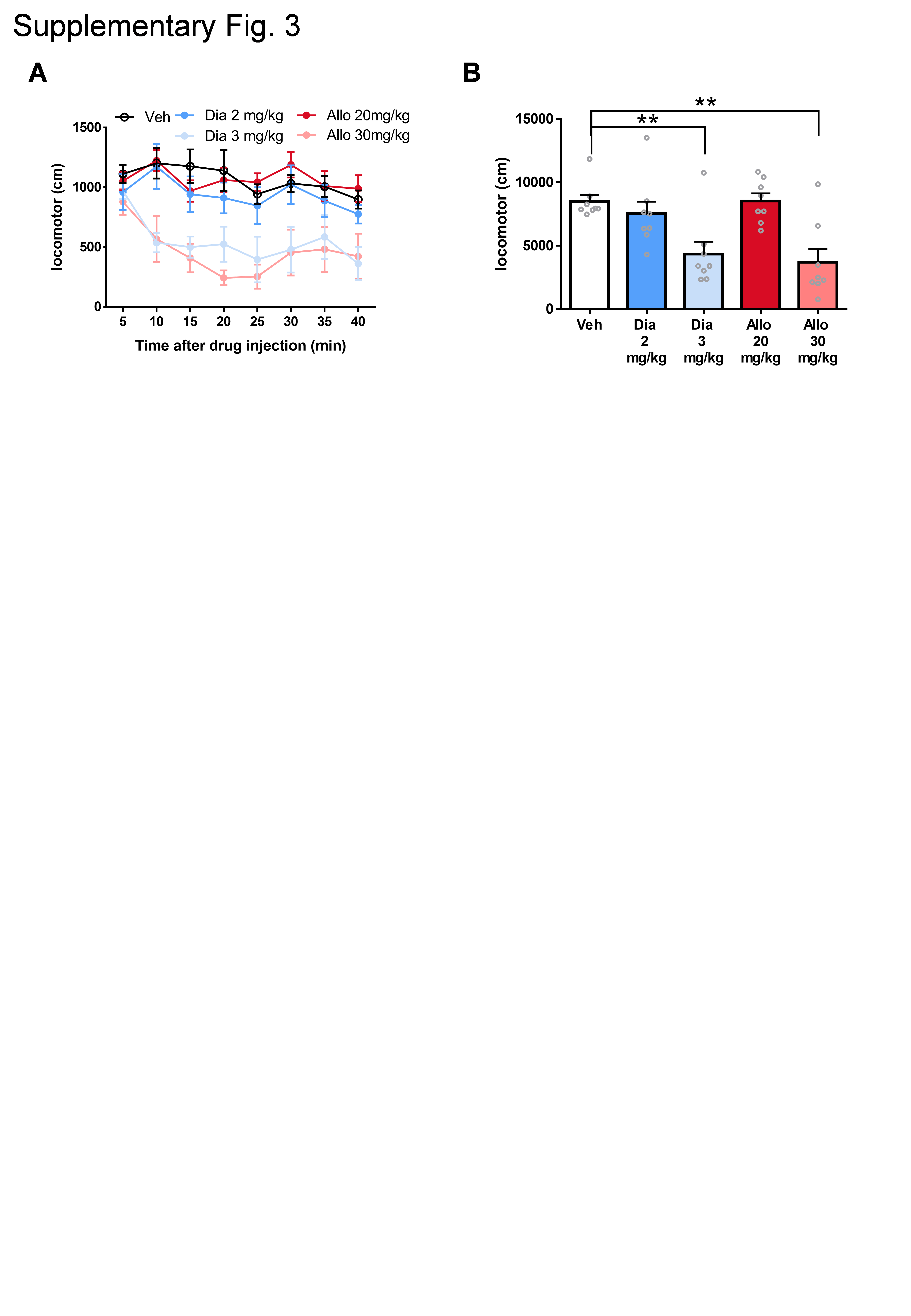

Supplement: Supplementary file 3 [file Image_3.tif]

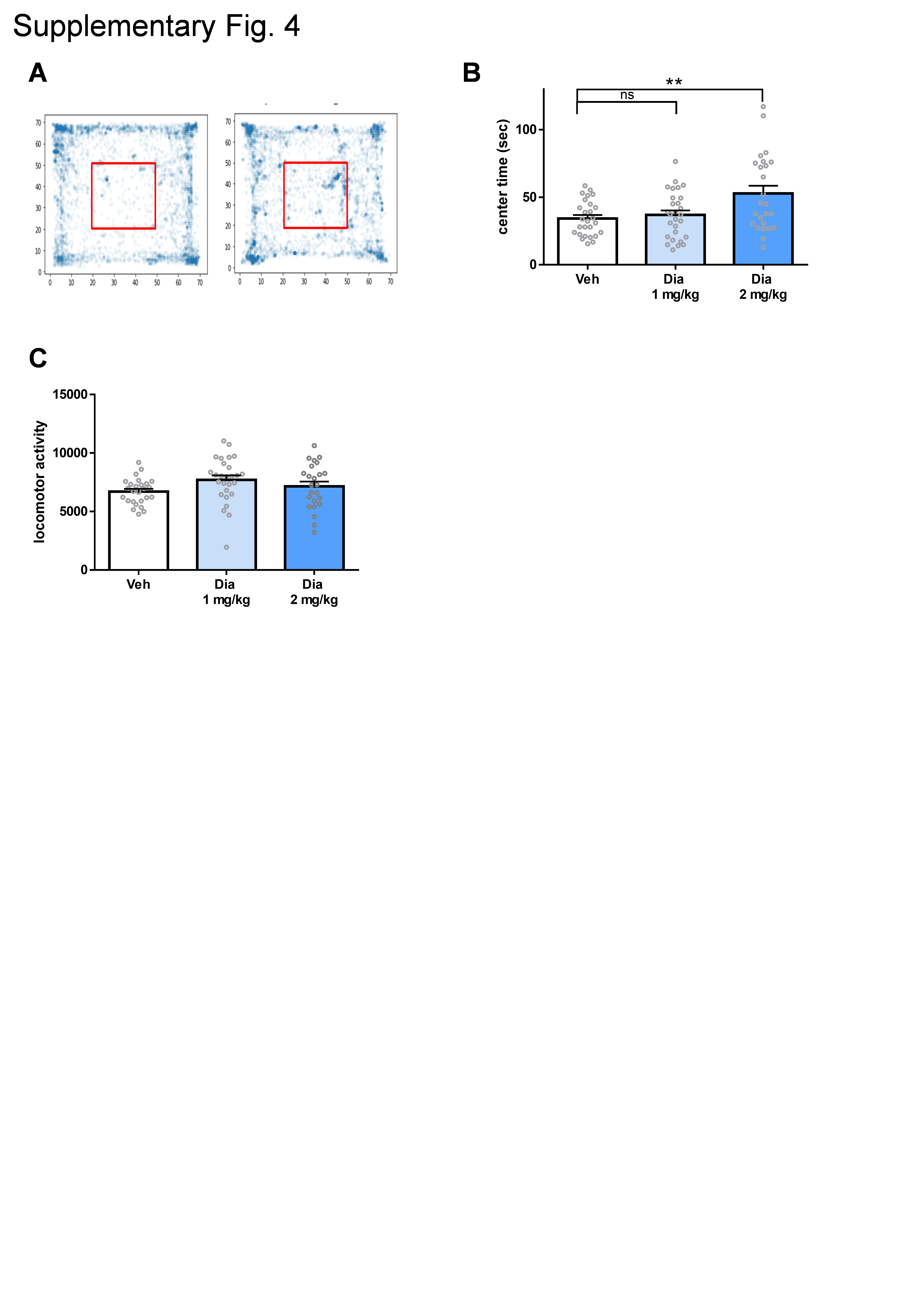

Supplement: Supplementary file 4 [file Image_4.tif]

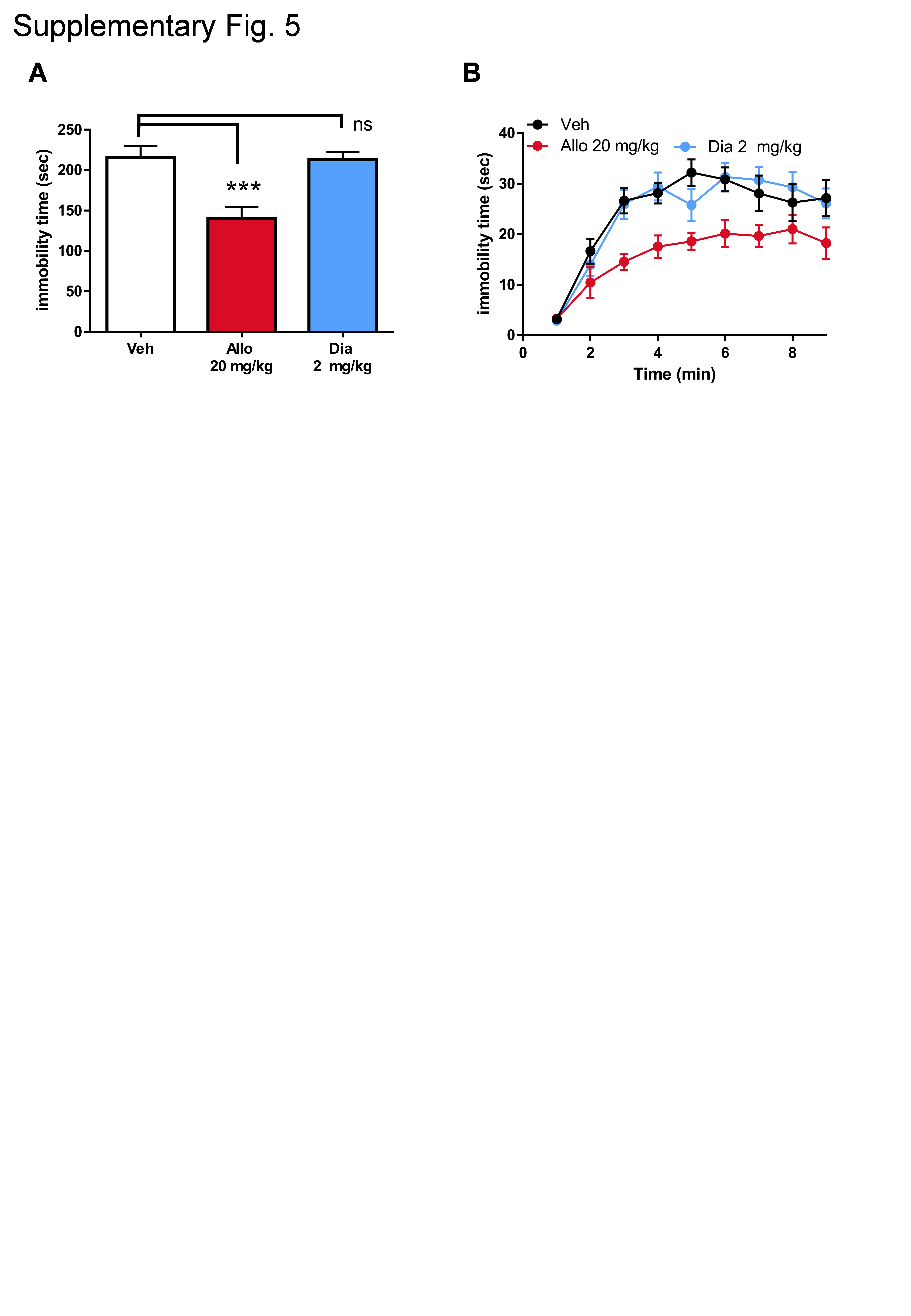

Supplement: Supplementary file 5 [file Image_5.tif]

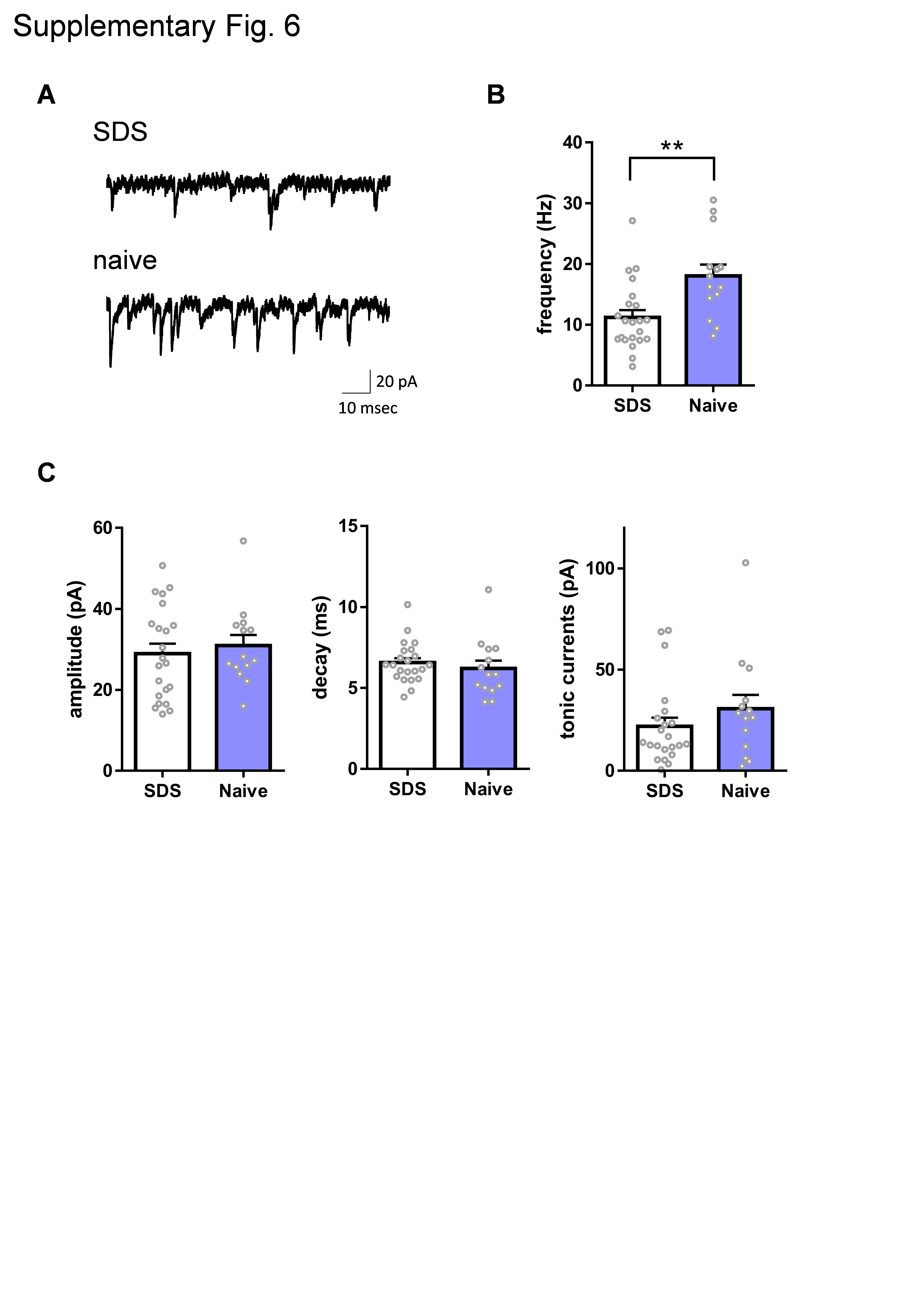

Supplement: Supplementary file 6 [file Image_6.tif]
